# Exploring the functions of NKG7 in CD4^+^ and CD8^+^ T cells during malaria

**DOI:** 10.1371/journal.ppat.1013528

**Published:** 2026-09-08

**Authors:** Teija C-M. Frame, Fabian de Labastida Rivera, Nicholas L. Dooley, Jessica A. Engel, Jinrui Na, Yulin Wang, Luzia Bukali, Susanna S. Ng, Patrick T. Bunn, Lynette Beattie, Gunter Hartel, Timothy William, Matthew J. Grigg, Nicholas M. Anstey, James S. McCarthy, Bridget E. Barber, Michelle J. Boyle, Christian R. Engwerda

**Affiliations:** 1 QIMR Berghofer Medical Research Institute, Brisbane, Australia; 2 University of Queensland, Brisbane, Australia; 3 Burnet Institute, Melbourne, Australia; 4 University of Bonn, Bonn, Germany; 5 Department of Infectious Diseases, The University of Melbourne at the Peter Doherty Institute for Infection and Immunity, Melbourne, Australia; 6 Menzies School of Health Research, Charles Darwin University, Darwin, Northern Territory, Australia; 7 Infectious Diseases Society Kota Kinabalu Sabah-Menzies School of Health Research Program, Kota Kinabalu, Sabah, Malaysia; 8 Subang Jaya Medical Centre, Selangor, Malaysia; University of Georgia, UNITED STATES OF AMERICA

## Abstract

Malaria, caused by *Plasmodium* parasites, is a significant global health issue. CD4^+^ and CD8^+^ T cells are important for immunity against *Plasmodium* infections, but the specific roles of many immune-related effector molecules in T cells remain poorly defined. Here, we investigated the function of NK cell granule protein 7 (NKG7) in T cells during malaria, focusing on its role in CD4^+^ and CD8^+^ T cells in *Plasmodium* blood-stage responses. In a non-lethal malaria model, NKG7 regulated the development of pro-inflammatory T helper 1 (Th1), IL-10-producing type 1 regulatory (Tr1), and T follicular helper cell responses. In a model of cerebral malaria, NKG7 had a cell-intrinsic role in regulating perforin and granzyme B expression, as well as Tr1 cell development. Human investigations involving peripheral blood mononuclear cells from volunteers participating in controlled human *P. falciparum* malaria infection studies, as well as endemic country patients with *P. falciparum* and *P. vivax* malaria, corroborated these findings. High NKG7 expression in T cells from *Plasmodium*-infected humans was observed, as well as differences in NKG7 expression based on the infecting *Plasmodium* species. NKG7 expression was associated with both cytotoxic and non-cytotoxic T cells, indicating varied functions following infection. These results advance our understanding about NKG7’s role in T cell-mediated malaria immunity and suggest potential for targeting NKG7 to improve outcomes following *Plasmodium* infection.

## Author summary

Malaria remains a major global health burden, and effective immunity depends on coordinated responses by CD4⁺ and CD8 ⁺ T cells. However, the specific molecules that enable these T cells to control infection or contribute to pathology are not fully understood. Here, we investigate the role of NK cell granule protein 7 (NKG7), a molecule associated with cytotoxic function, in T cell responses during malaria. Using mouse models of non-lethal and cerebral malaria, we show that NKG7 is required for optimal development of key CD4 ⁺ T cell subsets, including Th1, IL-10-producing Tr1, T follicular helper, and cytotoxic CD4 ⁺ T cells. In human controlled malaria infection studies and in patients with *Plasmodium falciparum* or *P. vivax* malaria, we identify multiple NKG7-expressing CD4⁺ and CD8 ⁺ T cell populations with species-specific patterns. These findings reveal diverse roles for NKG7 in malaria immunity and highlight its potential as a target to modulate host responses.

## Introduction

Malaria has a profound impact on human health, with 263 million cases causing an estimated 597,000 deaths in 83 endemic countries in 2023 [1]. The COVID-19 pandemic intensified malaria incidence, leading to an estimated 13.4 million additional cases from 2019 to 2021 [2], with recent global events further threatening the stability of malaria control programs. Africa bears the heaviest burden, accounting for 94% of malaria cases and 95% of malaria deaths, with children under five being the most affected [1]. *Plasmodium* parasites, particularly *P. falciparum*, and to a lesser extent *P. vivax*, cause most disease through complex interactions between parasites and human hosts [1,3]. Animal models, such as rodent malaria infections, help advance understanding of disease pathogenesis and immunity [4–6], complementing *ex vivo* studies of clinical samples from malaria patients. Both animal and human studies have highlighted key roles for both CD4^+^ and CD8^+^ T cells in pathogenesis and immunity in malaria [3,7]. An improved understanding about these cells will be needed for the development of second-generation malaria vaccines.

*Plasmodium*-specific CD4 ⁺ T cells are crucial mediators of protective immunity against malaria following natural exposure or vaccination [8–11], encompassing subsets such as T helper 1 (Th1), type I regulatory (Tr1), and T follicular helper (Tfh) cells that play important roles in both liver and blood stages of infection [3,12]. Th1 cells, characterized by IFNγ production, are essential for controlling parasite burdens by activating macrophages and influencing B cell responses [13–18], though they can also contribute to malaria pathology by promoting atypical memory B cell development, inflammation and anaemia [19,20]. Tr1 cells, which produce IL-10, help regulate inflammation but may hinder effective parasite control, with their presence linked to both higher parasitemia and reduced severe disease risk, highlighting their complex role in immune modulation [3,12,21–23]. Tfh cells are vital for fostering protective antibody responses by supporting the maturation of germinal centre (GC) B cells, leading to somatic hypermutation and the production of high affinity antibodies that target various *Plasmodium* lifecycle stages through mechanisms including antibody-dependent cell cytotoxicity and phagocytosis [24–29]. Overall, these CD4 ⁺ T cell subsets orchestrate a balance between effective immunity and immune regulation to manage *Plasmodium* infections.

Along with CD4 ⁺ T cells, *Plasmodium*-specific CD8 ⁺ T cells are detected in people with natural exposure to malaria [30], and after vaccination [31]; these show responses against sporozoite, liver, and blood-stage antigens [32,33]. These cells can be important for liver-stage immunity, where they may prevent progression to symptomatic blood-stage infection [8,34,35]. Antigen-presenting cells in lymph nodes and the spleen prime CD8 ⁺ T cells [36–38], which employ both cytolytic- and cytokine-mediated mechanisms, including IFNγ, TNF, perforin, granzyme and granulysin production, to protect against liver-stage *Plasmodium* [39–41]. Liver-resident CD8 ⁺ T cells, generated by specific vaccination strategies in pre-clinical malaria models, form a protective niche in hepatic sinusoids [42–45]. While CD8 ⁺ T cells have limited roles in the blood stage of *P. falciparum* due to the lack of MHC class I on mature erythrocytes [46], responses against blood-stage antigens in *P. vivax* infection can eliminate infected reticulocytes [47]. However, in experimental cerebral malaria (ECM) in mice, CD8 ⁺ T cells may drive brain pathology through granzyme B, perforin, and IFNγ-mediated mechanisms [48,49], targeting vascular endothelial cells cross-presenting parasite antigens [50–52].

Mechanisms of CD4^+^ and CD8^+^ T cell activation and differentiation in malaria are incompletely understood. We previously identified key roles for the natural killer cell protein 7 (NKG7) expression by CD8^+^ T cells for driving inflammation in ECM by facilitating CD107a translocation and cellular killing [53]. Initially identified as NK cell-specific in 1990 [54], the *NKG7* gene encodes a 148-amino acid protein with cytotoxic granule membrane localization in NK cells, which translocates to the plasma membrane upon cell activation [55–59]. NKG7 is also highly expressed in cytotoxic CD4⁺ and CD8 ⁺ T cells [60–64], correlating with Th1 cell polarization [65–67], and implicated in immune responses against infections like HIV, cytomegalovirus and COVID-19 [60,61,68]. Its expression is also linked to cytotoxic functions in cancer and autoimmune disorders [69–76], making NKG7 a significant biomarker and potential target in transplant medicine, cancer, and immune-mediated inflammatory diseases. Expanding on our previous work [53,77], here we investigate the role of CD4^+^ T cell NKG7 in non-lethal and lethal experimental malaria models, as well as NKG7 expression by CD4^+^ and CD8^+^ T cells in clinical samples from humans infected with *P. falciparum* and *P. vivax*.

## Results

### NKG7 has a protective role during non-lethal experimental malaria following *Plasmodium yoelii* 17XNL infection

**Fig 1:**
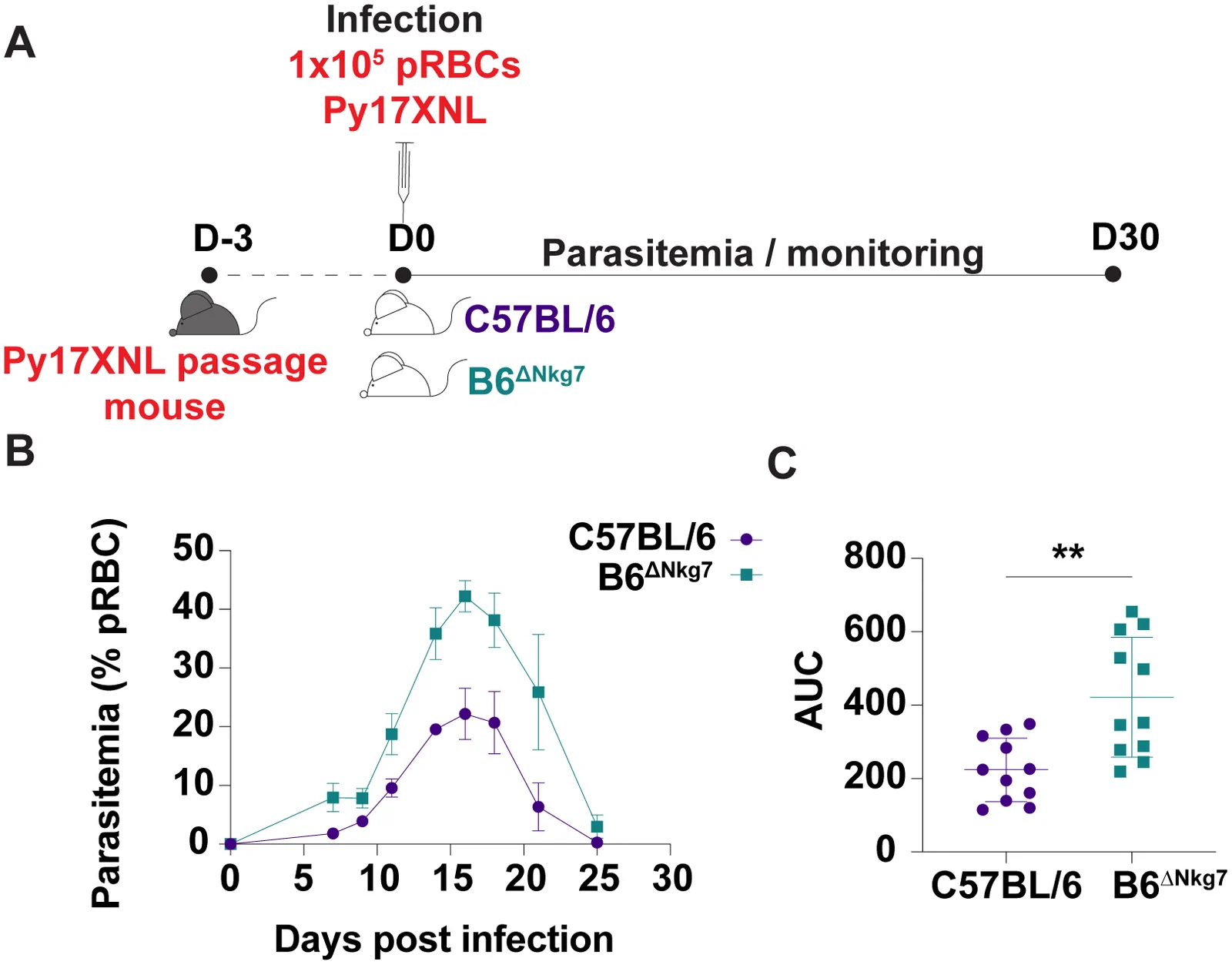
NKG7 has a protective role during non-lethal experimental malaria caused by *Plasmodium yoelii* 17XNL (*Py*17XNL) infection. **(A)** C57BL/6 and B6.*Nkg7*^-/-^ (B6^∆*Nkg7*^) mice were infected with non-lethal *Py*17XNL. Layout for the experimental timeline. **(B)** Frequency of parasitised RBCs (pRBC) in peripheral blood of B6 and B6^∆*Nkg7*^mice **(B)**. **(C)** Area Under the Curve (AUC), as a measure of parasite burden over the course of infection. Data is pooled from two independent experiments. A Mann-Whitney test was used to test for statistical significance, where ** P < 0.01. n = 11 per group.

We previously showed a key role for NKG7 expressed by CD8^+^ T cells in the pathogenesis of ECM caused by infection of C57BL/6 mice with *P. berghei* ANKA (*Pb*A) [53]. A key limitation of the ECM model is the acute and lethal nature of *Pb*A infection. Therefore, to investigate the role of NKG7 in parasite control and the development of immunity against malaria, an experimental mouse model of non-lethal, resolving malaria caused by *P. yoelii* (*Py*17XNL) infection was employed. We first infected *Nkg7*-deficient (B6^Δ*Nkg7*^) and wild-type (WT) control C57BL/6 mice, and measured the progression of blood parasitemia through the course of infection (Fig 1A). Higher frequencies of parasitised red blood cells (pRBCs) were observed in *Nkg7-*deficient mice, compared to their WT counterparts (Fig 1B). To assess the overall parasite burden more comprehensively, the area under the curve (AUC) was calculated from blood parasitemia data points, revealing a significant increase in parasite burden among *Nkg7*-deficient mice (*P* < 0.05; Fig 1C). These results indicate an important role for NKG7 in the control of *Py*17XNL infection.

### The role of NKG7 in Th1 and Tfh cell responses during non-lethal experimental malaria following *Plasmodium yoelii* 17XNL infection

**Fig 2:**
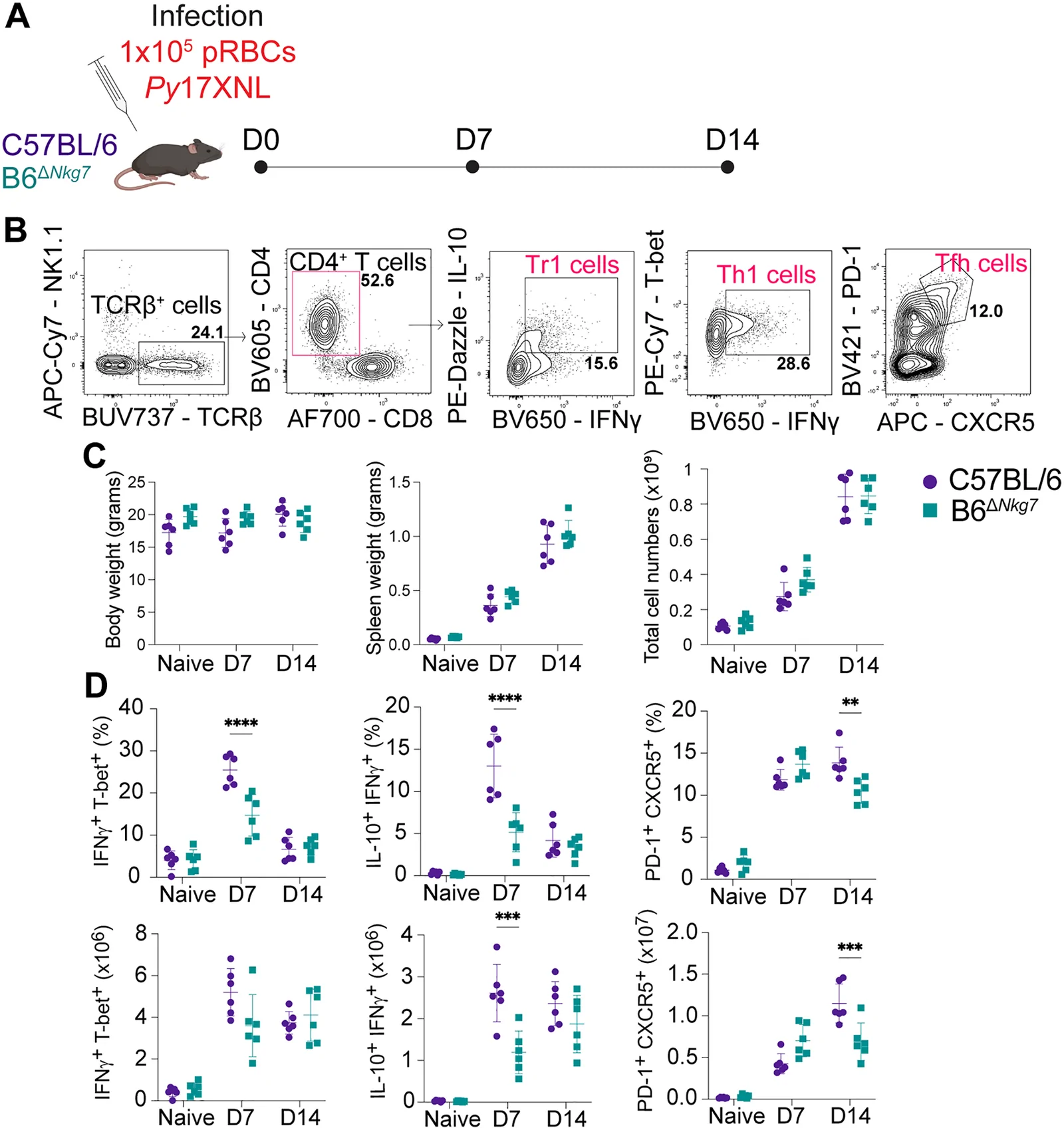
The role of NKG7 in CD4^+^ T cells during non-lethal experimental malaria caused by *Plasmodium yoelii* 17XNL (*Py*17XNL) infection. **(A)** Schematic showing the experimental timeline. Created in BioRender. Engwerda, C. (2026) https://BioRender.com/wxzptf9. **(B)** Gating strategy used to identify CD4^+^ T cell subsets. **(C)** Body and spleen weights of naïve and *Py*17XNL infected C57BL/6 and B6.*Nkg7*^-/-^ (B6^∆*Nkg7*^) mice. The right graph shows the total splenocyte numbers of naïve and infected C57BL/6 and B6^∆*Nkg7*^ mice. **(D)** Frequencies of IFNγ^+^ T-bet^+^ (Th1) cells and IFNγ^+^ IL-10^+^ (Tr1) cells as a percentage of TCRβ^+^ CD4^+^ T cells after stimulation with phorbol 12myristate 13-acetate and ionomycin. The corresponding numbers of these CD4^+^ T cell subsets in the spleen are also shown. The graphs on the right indicate frequencies of PD-1^+^ CXCR5^+^ (Tfh) cells as a frequency of TCRβ^+^ CD4^+^ T cells, and total number shown below. Data are from one representative experiment of two performed. A two-way ANOVA with Sidak’s multiple comparisons test was used to calculate statistical significance, where ** P < 0.01, *** P < 0.001, **** P < 0.0001. n = 6 mice per group.

To investigate whether NKG7 played a role in CD4^+^ T cell development in this malaria model, Th1 and Tfh cell responses were examined at two specific time points during infection. Both *Nkg7-*deficient mice and WT mice were infected with *Py*17XNL, and flow cytometry analysis was conducted on days 7 and 14 post-infection (p.i.) (Fig 2A and 2B). We found no significant differences in body weight, development of splenomegaly or increase in spleen cell numbers between *Nkg7*-deficient mice and WT mice at either time point during infection (Fig 2C). Upon treatment with PMA and ionomycin in the presence of monensin, significantly decreased frequencies of Th1 (IFNγ^+^ T-BET^+^) and Tr1 (IFNγ^+^ IL-10^+^) cells were observed in *Nkg7*-deficient mice at day 7 p.i., when compared to WT mice (*P* < 0.05; Fig 2D). Additionally, significantly reduced frequencies and numbers of Tfh (PD-1^+^ CXCR5^+^) cells were noted at day 14 p.i. in *Nkg7*-deficient mice compared to their WT counterparts (*P*< 0.05; Fig 2D). A limitation with this data was no FoxP3 staining of CD4^+^ T cells, meaning that natural regulatory T (Treg) cells were not excluded from analysis. To determine whether they did contribute to the above results, we repeated these experiments and employed FoxP3 staining of CD4^+^ T cells (S1 Fig). These data showed no difference in the frequency or number of Treg cells between *Nkg7*-deficient mice and WT mice following *Py*17XNL infection (S1B-D Fig). Furthermore, no cytokine production was detected from these cells during infection (S1B and S1D Fig). Thus, these data suggest a potential role for NKG7 in Th1 and Tr1 cell development during the first week of *Py*17XNL infection, as well as in Tfh cell development during the second week of *Py*17XNL infection.

These findings were supported when we examined the role of NKG7 in antigen-specific CD4^+^ T cells activation and differentiation in the lethal ECM model using PbT-II T cells. These are MHC-II-restricted TCR transgenic T cells which recognise a peptide derived from the *PbA* Hsp90 protein which is expressed by all rodent *Plasmodium* species [78]. To investigate cell-intrinsic roles for NKG7 in these cells, equal numbers of control (PbT-II^WT^) and *Nkg7*-deficient (PbT-II^Δ*Nkg7*^) cells were adoptively transferred into congenic *Ptprca* (CD45.1) recipient mice prior to *Pb*A infection. Cellular analysis was performed when the mice developed ECM at day 5 p.i. (S2A-B Fig). After treatment with PMA and ionomycin, in the presence of monensin, despite a small, but significant increase in IFNγ^+^ PbT-II^Δ*Nkg7*^ cells, there was a decrease in Tr1 (IFNγ^+^ IL-10^+^) cell frequencies, compared with PbT-II^WT^ cells from the same animals (*P* < 0.05; S2C Fig). PbT-II^Δ*Nkg7*^ cells also exhibited significantly lower frequencies of granzyme B-producing cells when compared to PbT-II^WT^ cells (*P* < 0.05), but no difference in CD107a expression (S2C Fig), as previously observed for *Nkg7*-deficient CD8^+^ T cells [53]. We next examined their functional potential by assessing expression of different co-inhibitory receptors, but no differences in the expression of these molecules by PbT-II^WT^ and PbT-II^Δ*Nkg7*^ Tr1 cells was found (S2D Fig). However, similar to granzyme B, a significant reduction in the frequency of perforin-producing PbT-II^Δ*Nkg7*^ cells was observed, when compared to the PbT-II^WT^ cells (*P* < 0.05; S2E Fig). Together, these results identify roles for NKG7 in CD4^+^ T cells during *Pb*A infection in perforin and granzyme B expression, as well as the development of Tr1 cells.

### Increased *Nkg7* expression by Th1 and Tr1 cells during *Plasmodium yoelii* 17XNL infection

**Fig 3:**
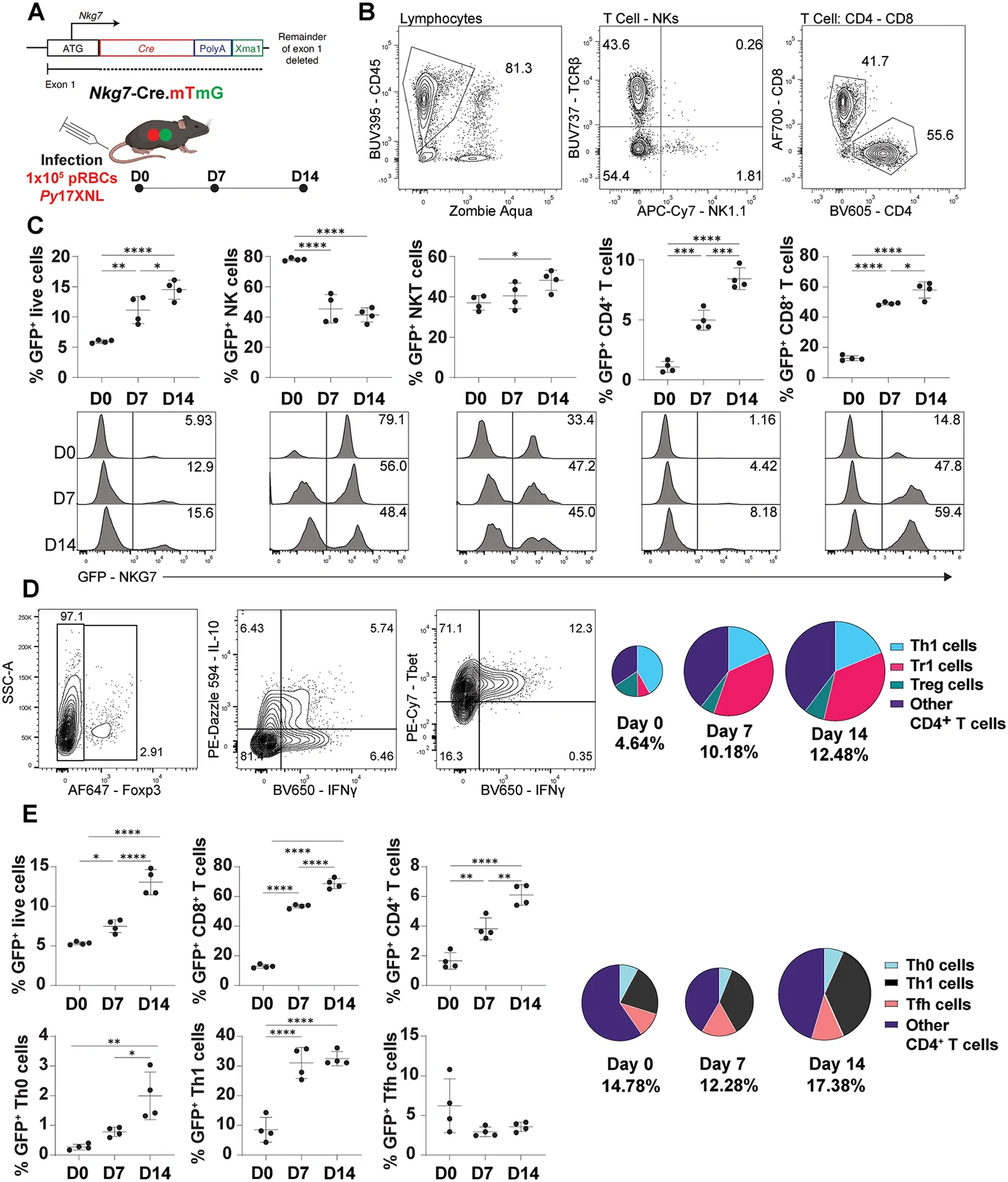
*Nkg7* expression increases in Th1 and Tr1 cells during non-lethal *Plasmodium yoelii* 17XNL (*Py*17XNL) infection. **(A)** Schematic showing the experimental timeline for B6.*Nkg7*-reporter mice (*Nkg7*-cre x mT/mG) infected with *Py*17XNL. Created in BioRender. Engwerda, C. (2026) https://BioRender.com/wxzptf9. **(B)** Gating strategy used to identify lymphoid cell subsets. **(C)** Frequencies of GFP^+^ (*Nkg7*^+^) in lymphoid cell subsets, as indicated, during *Py*17XNL infection. **(D)** Representative gating strategy and frequencies of GFP^+^ (*Nkg7*^+^) CD4^+^ IFNγ^+^ T-bet^+^ (Th1) cells, IFNγ^+^ IL-10^+^ (Tr1) cells, Foxp3^+^ regulatory T (Treg) cells and the remaining CD4^+^ T cells after stimulation with phorbal 12myristate 13-acetate and ionomycin, shown as a pie chart. The size of the compartments within the pie chart indicates the percentage of the CD4^+^ T cell subsets specified, within the GFP^+^ CD4^+^ T cell population. **(E)** Frequencies of all GFP^+^ (*Nkg7*^+^) cells, CD8^+^ T cells and CD4^+^ T cells during *Py*17XNL infection directly *ex vivo*. CD4^+^ T cells were further divided into CXCR6^-^ T-bet^-^ (Th0) cells, CXCR6^+^ T-bet^+^ (Th1) cells, PD-1^+^ CXCR5^+^ (Tfh) cells and the remaining CD4^+^ T cells, shown as a pie chart. The size of the compartments within the pie chart indicates the percentage of the CD4^+^ T cell subsets specified, within the GFP^+^ CD4^+^ T cell population. Statistical significance was assessed by ordinary one-way ANOVA with Tukey’s multiple comparisons test, where * P < 0.05, ** P < 0.01, *** P < 0.001, **** P < 0.0001. n = 4 naïve or infected mice. Data are from one representative experiment of two performed.

To characterise the cellular expression of NKG7 during *Py*17XNL infection, a *Nkg7*-transcriptional reporter mouse was utilised (Fig 3A-B). In these mice, cells in which *Nkg7* transcription is initiated switch from expressing TdTomato (red) to GFP (green) [53]. In the naïve state, the majority of NK cells (Fig 3C) expressed GFP, but this expression significantly decreased following infection (*P* < 0.05), as previously reported in experimental visceral leishmaniasis [53]. Conversely, the frequency of GFP^+^ cells increased significantly in NKT, CD4^+^ and CD8^+^ T cell subsets during the infection (*P* < 0.05; Fig 3C). The frequency of GFP-expressing CD4^+^ T cells increased significantly from the naïve state to day 7 p.i. and was sustained at day 14 p.i. (*P* < 0.001 and *P* < 0.0001, respectively; Fig 3C). To further investigate this increase, we next examined *Nkg7* expression within distinct CD4^+^ T cell subsets. Following activation with PMA and ionomycin, Th1 cells dominated in the naïve state, while Tr1 cells became dominant at days 7 and 14 p.i. (Fig 3D). The compartment representing other CD4^+^ T cells included cells that were neither Th1 cells, Tr1 cells, nor Treg cells. When analysing T cell subsets directly *ex vivo*, without further stimulation, a similar trend emerged with significantly increasing frequencies of GFP^+^ cells during the infection in CD4^+^ and CD8^+^ T cells (*P* < 0.05; Fig 3E), as well as in the CD4^+^ T cell subsets, including Th0 cells (T-BET^-^ CXCR6^-^), Th1 cells (T-bet^+^ CXCR6^+^), and other CD4^+^ T cells (non-Tfh T-bet^+^ CXCR6^+^) (*P* < 0.05; Fig 3E). We were unable to detect IL-10-producing Tr1 cells without *ex vivo* activation with mitogens in this analysis. Interestingly, the frequency of GFP^+^ Tfh cells (PD-1^+^ CXCR5^+^) remained consistent between the naïve state and both infection time points (Fig 3E). These data suggest that NKG7 is expressed across multiple CD4^+^ T cell subsets with enrichment in Th1 and Tr1 cells following *Py*17XNL infection.

### A cell intrinsic role for NKG7 in the development of Th1, Tr1 and Tfh cells during *Plasmodium yoelii* 17XNL infection

**Fig 4:**
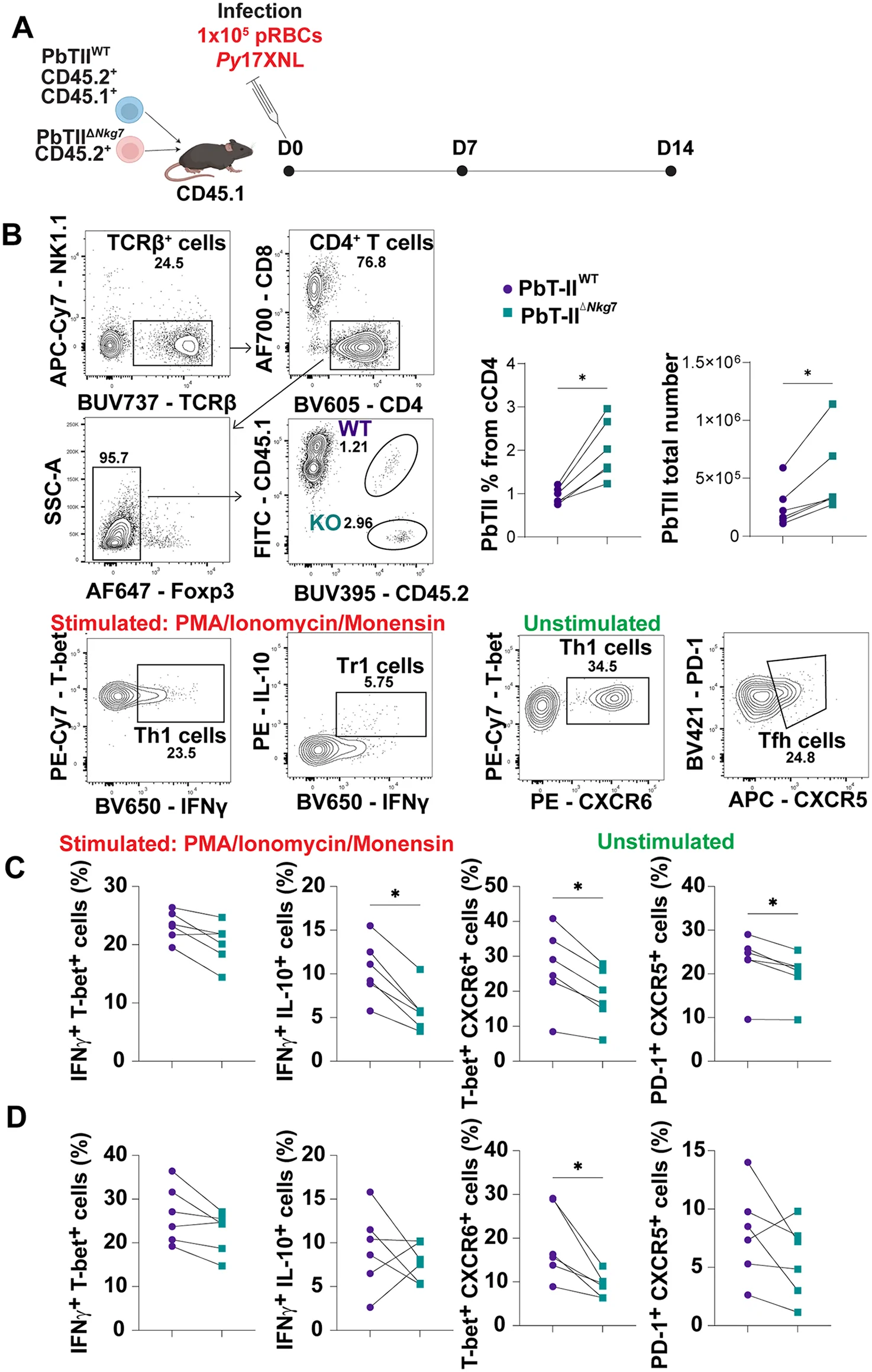
NKG7 has a cell intrinsic role in CD4^+^ T cell responses during non-lethal experimental malaria caused by *Plasmodium yoelii* 17XNL (*Py*17XNL) infection. **(A)** Experimental timeline for co-transfer of transgenic PbT-II^WT^ and PbT-II^Δ*Nkg*7^ cells into B6.CD45.1 mice prior to infection with *Py*17XNL. Created in BioRender. Engwerda, C. (2026) https://BioRender.com/wxzptf9. **(B)** Gating strategy used to identify and phenotype PbT-II cell subsets with or without stimulation with phorbol ester and ionophore. Frequency of PbT-II cells, relative to conventional (cCD4), as well as total PbT-II cell numbers on day 7 post-infection. **(C)** Frequencies of co-transferred transgenic PbT-II^WT^ and PbT-II^Δ*Nkg*7^ IFNγ^+^ T-bet^+^ (Th1) cells and IFNγ^+^ IL-10^+^ (Tr1) cells as a proportion of parental PbT-II cells (CD45.1/2+ (PbT-II^WT^) and CD45.2+ (PbT-II^Δ*Nkg*7^)) from the spleen at day 7 post infection after stimulation with phorbol ester and ionophore (two left panels). Frequencies of co-transferred transgenic PbT-II^WT^ and PbT-II^Δ*Nkg*7^ T-bet^+^ CXCR6^+^ cells and PD-1^+^ CXCR5^+^ (Tfh) cells as a proportion of parental PbT-II cells, as above, from the spleen at day 7 post infection *ex vivo* (no further stimulation; two right panels). **(D)** Frequency of the same PbT-II cells as above at day 14 post infection. A Wilcoxon test was used to define statistical significance, where * P < 0.05, n = 6 mice per group. Data are from one representative experiment of two performed.

To investigate whether NKG7 played a cell-intrinsic role in CD4^+^ T cells in the non-lethal malaria model, equal numbers of PbT-II^WT^ and PbT-II^Δ*Nkg7*^ cells were adoptively transferred into congenic *Ptprca* (CD45.1) recipient mice prior to *Py*17XNL infection. Cellular analyses were performed at both day 7 and day 14 p.i. to evaluate Th1, Tr1 and Tfh cell responses, with FoxP3 included in the staining panels as an exclusion marker (Fig 4A and 4B). In this adoptive transfer model where PbT-II^WT^ and PbT-II^Δ*Nkg7*^ cells were primed and expanded in the same host, we found greater expansion of PbT-II^Δ*Nkg7*^ cells, relative to PbT-II^WT^ cells during the first seven days of infection (Fig 4B), suggesting a CD4^+^ T cell cell intrinsic role for NKG7 in cell expansion not observed in *Nkg7*-deficient mice (Fig 2D). However, at day 7 p.i., following treatment with PMA and ionomycin in the presence of monensin to assess cellular potential, no discernible differences were observed in the frequencies of Th1 cells (IFNγ^+^ T-bet^+^) between the two PbT-II populations (Fig 4C). We did observe a significant reduction in the frequencies of PbT-II^Δ*Nkg7*^ Tr1 cells (IFNγ^+^ IL-10^+^) (*P* < 0.05; Fig 4C). In unstimulated cells analysed *ex vivo*, a significant decrease was noted in PbT-II^Δ*Nkg7*^ Tfh cell (PD-1^+^ CXCR5^+^) frequencies, as well as PbT-II^Δ*Nkg7*^ Th1 (T-bet^+^ CXCR6^+^) cells (*P* < 0.05; Fig 4C). CXCR6 was used because it was previously identified as a robust Th1 cell marker of PbT-II cells without the need to *ex vivo* stimulation [79,80]. At day 14 p.i., in unstimulated cells, significant differences were only observed in Th1 (T-bet^+^ CXCR6^+^) cells (*P* < 0.05; Fig 4D). Taken together, these data provide support for a cell-intrinsic role of NKG7 in the development of Th1, Tr1 and Tfh cells at day 7 p.i. and Th1 cells at day 14 p.i..

To test whether the above results could be caused by NKG7-dependent changes in proliferation or apoptosis, an early time course assessment of PbT-II^WT^ and PbT-II^Δ*Nkg7*^ cell proliferation, activation and apoptosis was performed. The *PbA* model rather than the *Py*17XNL model was chosen to address this question because it provides more rapid and robust PbT-II cell proliferation over a short timeframe. Equal numbers of PbT-II^WT^ and PbT-II^Δ*Nkg7*^ cells were adoptively transferred into congenic *Ptprca* (CD45.1) recipient mice prior to *Pb*A infection and cellular analysis performed each day, starting from the day of infection to day 5 p.i. (S3A-B Fig). Expansion of both PbT-II cell populations was observed at day 4 p.i. (S3C Fig), although the frequency of PbT-II^Δ*Nkg7*^ cells, was reduced at days 4 and 5 p.i., relative to PbT-II^WT^ cells. Analysis of CTV dilution revealed limited differences between PbT-II^WT^ and PbT-II^Δ*Nkg7*^ cells, and both populations had undergone approximately six rounds of cell division by day 3 p.i. (S3D Fig), with most cells CTV-negative by day 4 p.i.. In addition, few differences in CD4^+^ T cell-specific activation markers between PbT-II^WT^ and PbT-II^Δ*Nkg7*^ cells were seen, although an early increase in the frequencies of CD69^+^ and CD25^+^ PbT-II^Δ*Nkg7*^ cells, as well as small changes in CD11a expression, relative to PbT-II^WT^ cells was noted one day after infection for CD69 and CD25, and throughout infection for CD11a (S3E Fig). Apoptosis analysis of PbT-II^WT^ and PbT-II^Δ*Nkg7*^ cells demonstrated limited differences in frequencies of early apoptotic (Annexin V^+^ 7-AAD^-^) PbT-II^Δ*Nkg7*^ cells, compared to PbT-II^WT^ cells, and no differences in frequencies of cells that were in late apoptosis or already dead (Annexin V^+^ 7-AAD^+^) (S3F Fig). Together, these results show limited roles for NKG7 in proliferation and apoptosis following *Pb*A infection, although the reduced expansion of PbT-II^Δ*Nkg7*^ cells, relative to PbT-II^WT^ cells at days 4 and 5 p.i., accompanied by limited changes in proliferation and apoptosis, indicate potential model and timepoint limitations compared with *Py*17XNL infection, possibly reflecting tissue sequestration of CD4^+^ T cells in the former model.

### The expression of NKG7 during controlled human infection studies with *Plasmodium falciparum*

We next examined the pattern of NKG7 expression by human immune cells during malaria. To measure NKG7 expression in human peripheral blood mononuclear cells (PBMCs), we first tested the specificity of a previously reported monoclonal antibody [58]. We employed HEK293 cells transfected with human *NKG7-GFP* and the NK cell line NK92, for which CRISPR/cas9 was employed to delete *NKG7*. We readily detected NKG7 in *NKG7*-transfected HEK293 cells, but not in control cells, as well as NK92 wild-type cells, but not cells depleted of *NKG7*, where expression was significantly diminished at both mRNA and protein levels (S4 Fig). Thus, this anti-NKG7 antibody was included in flow cytometry antibody panels for further investigations.

**Fig 5:**
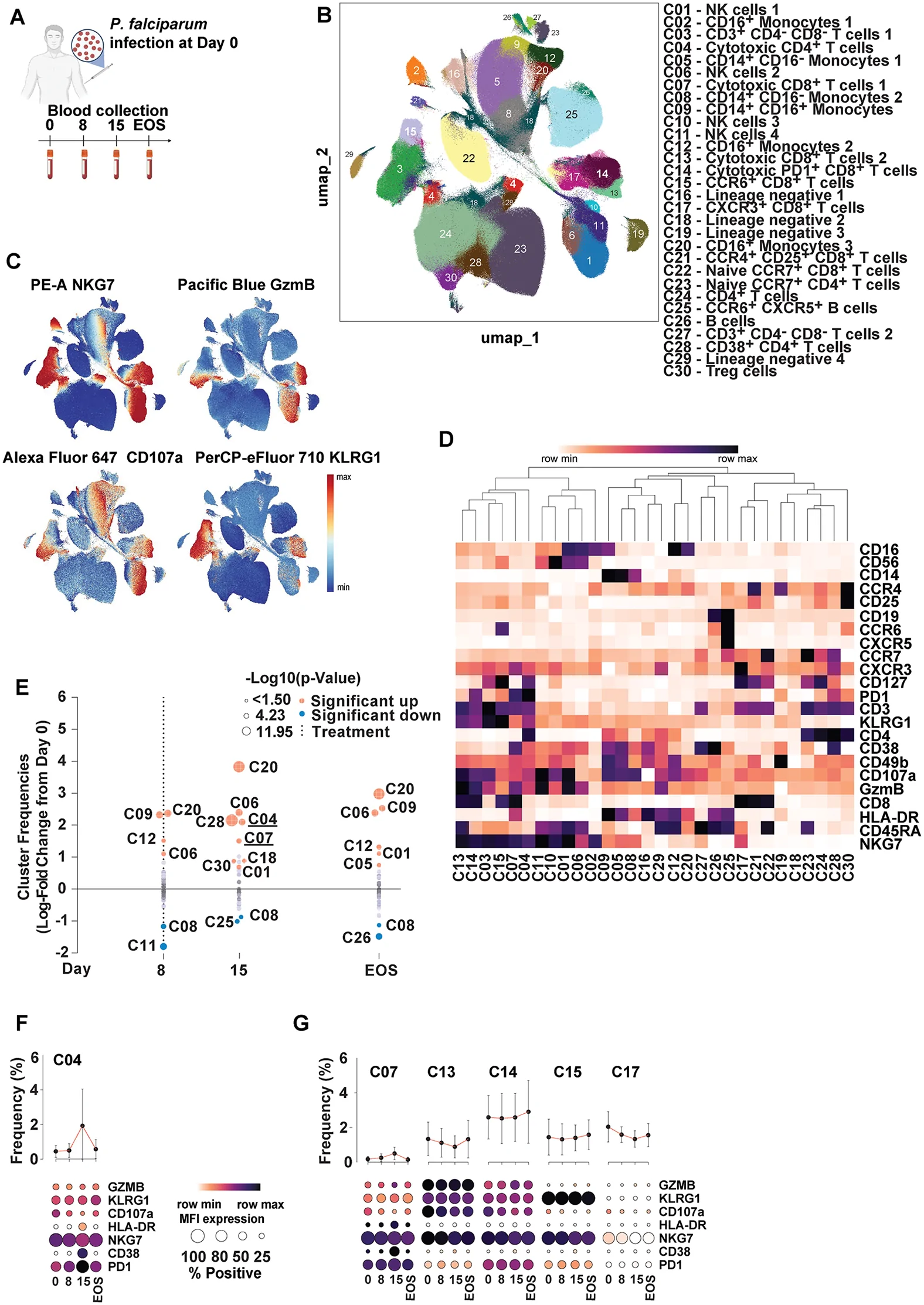
NKG7 expression correlated with cytotoxic and activated cells during controlled human malaria infection (CHMI). **(A)** Schematic showing the experimental design for CHMI studies; malaria-naïve subjects were infected with blood-stage *Plasmodium falciparum* 3D7 followed by treatment with an anti-parasitic trial drug, 8-9 days after infection. Peripheral blood mononuclear cells (PBMCs) were isolated from blood samples that were collected for analysis before infection (day 0), pre-treatment (day 8 post infection (p.i.), prior to drug administration), post anti-parasitic treatment (day 15 p.i.) and at the end of the study (EOS). Created in BioRender. Engwerda, C. (2026) https://BioRender.com/wxzptf9. **(B)** Cryopreserved PBMCs were thawed and immunophenotyped by spectral flow cytometry. Post-acquisition analysis was conducted using OMIQ software for dimensionality reduction using unsupervised FLOWSOM clustering that yielded 30 clusters. **(C)** Mean expression of cytotoxic markers NKG7, granzyme B (GzmB), CD107a, and KLRG1 are also shown separately on the UMAP. **(D)** Expression of cell phenotyping markers were measured, and mean fluorescence intensity (MFI) of staining presented as a heatmap. n = 31 volunteer samples, day 0 (n = 8), day 8 (n = 8), day 15 (n = 8), EOS (n = 7). **(E)** PBMCs were divided into 30 clusters and clusters that were significantly different in abundance between day 0 and 8, day 0 and 15, and day 0 and end of study (EOS) are indicated with red circles for significant increase and blue for significant decrease (significance assessed using EdgeR). T cell clusters of interest are underlined. **(F)** Frequency (top) and Dot plots (bottom) depicting Cluster change, expression patterns and frequency of positive events for cytotoxic and activation markers in a CD4^+^ T cell cluster and **(G)** CD8^+^ T cell clusters for the duration of the CHMI trial.

We next examined blood from healthy, malaria-naïve volunteers participating in controlled human malaria infection (CHMI) studies with blood-stage *P. falciparum* [81]. This involved samples from 8 individuals (5/8 male, aged median 22, range 18–29); all were treated with an anti-parasitic drug 8–9 days after infection, when blood parasite level exceeded 5000 parasites/mL in most participants [82]. PBMCs were isolated from blood and examined before infection (day 0), at pre-treatment (day 8 p.i., prior to drug administration), after anti-parasitic treatment (day 15 p.i.) and at the end of the study (EOS; day 45 ± 2) (Fig 5A). Unsupervised dimensionality reduction using UMAP, followed by clustering of flow cytometry data, identified 30 distinct cell clusters, (Figs 5B and S5A). Cell clusters with high NKG7 expression also had high expression of the cytotoxic molecules GZMB, CD107a and KLRG1 (Fig 5C). We identified one CD4^+^ T cell cluster (C04) and five CD8^+^ T cell clusters (C07, C13, C14, C15 and C17) that had high expression of NKG7 (Fig 5D-G). The NKG7^+^ CD4^+^ T cell cluster (C04) displayed characteristics of an effector memory CD4^+^ T cell cluster (CD25^-^ CD127^+^ CCR7^-^) that expressed multiple activation markers, including CD49b, PD1, CD38, KLRG1, GZMB and CD107a (Fig 5D and F). The five NKG7^+^ CD8^+^ T cell clusters lacked CCR7 expression and were identified as likely effector memory T cell clusters with varying levels of CD45RA expression. Three of the NKG7^+^ CD8^+^ T cell clusters (C07, C13 and C14) showed a similar cytotoxic phenotype with different combinations of KLRG1, CD38, and PD1 expression. Cluster 17 appeared to be a less differentiated CD8^+^ T cell cluster as it lacked expression of cytotoxic, activation or exhaustion markers and had low expression of KLRG1 and CCR7. Cluster 15 showed differentiation from the other four NKG7^+^ CD8^+^ T cell clusters and expressed high levels of CCR6, CD56 and KLRG1 and lacked expression of cytotoxic molecules (Fig 5D and G), suggesting it may represent either innate-like CD8^+^ T cells [83] or non-cytotoxic memory-like CD8^+^ T cells [84]. Examination of the differential abundance of cell clusters after infection (day 0 to day 8, day 0 to day 15, day 0 to EOS), revealed a significant increase (*P* < 0.05) in the abundance of the one NKG7^+^ CD4^+^ T cell cluster (C04) and one of the NKG7^+^ CD8^+^ T cell clusters (C07) when day 0 (pre-infection) was compared to day 15 (15 days p.i., 7 days post-drug treatment) (Fig 5E).

Further analysis of samples at day 15 p.i., when T cell activation appeared to peak, revealed significantly increased expression of CD38, PD1 and HLA-DR within the NKG7^+^ CD4^+^ T cell cluster (C04) (*P* < 0.05; Figs 5F and S4B). However, significantly decreased NKG7 expression from day 0 to day 15 was observed within this population, mirroring a decrease in CD107a expression (*P* < 0.05), and supporting a role for NKG7 in degranulation of effector molecules (S5B Fig). Cluster 7, which was the only CD8^+^ T cell cluster that was significantly different in abundance from day 0 to day 15 p.i. (Fig 5E), had relatively high expression of CD38, HLA-DR and PD-1 (Fig 5G), suggesting a highly activated and exhausted cluster. Similar to the NKG7^+^ CD4^+^ T cell cluster C04, a significantly decreased expression of CD107a from day 0 to day 15 was also observed in all NKG7^+^ CD8^+^ T cell clusters (*P* < 0.05; Figs 5G and S5B Fig). Together, these data provide new insights into CD4^+^ and CD8^+^ NKG7-expressing T cells during CHMI with multiple T cell subsets identified, each with a different functional potential.

### Species-specific differences in NKG7-expressing T cells during uncomplicated *Plasmodium falciparum* and *P. vivax* malaria

**Fig 6:**
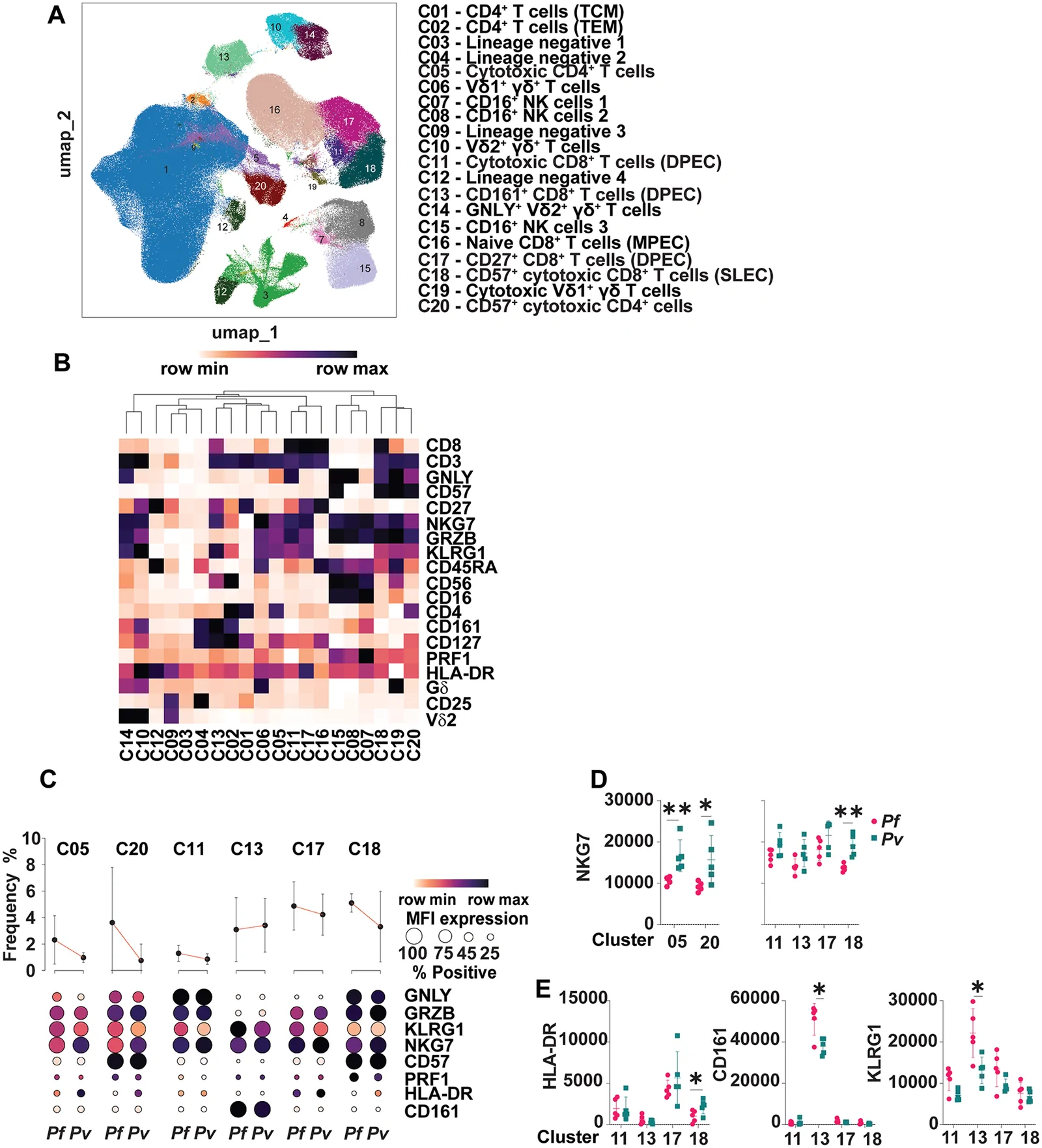
Differences in NKG7 expression during *P. falciparum* and *P. vivax* naturally acquired malaria. **(A)** Peripheral blood mononuclear cells (PBMCs) were isolated from blood samples collected from patients with naturally acquired, uncomplicated malaria at the Queen Elizabeth Hospital in Sabah, acute *P. falciparum* (*Pf*; n = 5) and acute *P. vivax* (*Pv*; n = 6). Post-treatment samples were also collected (n = 8). Cryopreserved PBMCs were thawed and immunophenotyped by spectral flow cytometry. Post-acquisition analysis was conducted using OMIQ software for dimensionality reduction using and unsupervised FLOWSOM clustering. **(B)** Expression of cell protein markers was measured and mean fluorescent intensity of staining presented as a heatmap. **(C)** Samples were divided into 20 clusters and the frequency of CD4^+^ and CD8^+^ T cell clusters are shown (top) and dot plots (bottom) depicting Cluster change, expression patterns and frequency of positive events for cytotoxic and activation markers compared between *Pf* and *Pv*. **(D)** NKG7 expression for CD4^+^ T cell clusters, and **(E)** HLA-DR, CD162 and KLRG1 expression for all CD8^+^ T cell clusters are shown. Significance was assessed by ordinary one-way ANOVA with Tukey’s multiple comparisons test, where * P < 0.05, n = 5-6 patient samples per group.

To test whether the NKG7 expression patterns observed in CHMI samples were also found during naturally acquired infection, we next investigated blood samples from patients admitted to a tertiary-referral hospital in Sabah, Malaysia, during 2010–2012, with uncomplicated *P. falciparum* or *P. vivax* malaria. Blood was collected at the time of presentation and one month post anti-parasitic treatment. These samples were classified into three groups: acute *P. falciparum* (*Pf*) infection (n = 5), acute *P. vivax* (*Pv*) infection (n = 6) and convalescence. The convalescence group included samples from 2 *Pf* and 6 *Pv* patients. Following analysis of flow cytometry data, 20 clusters were identified (Fig 6A), with the majority of NKG7-expressing clusters also expressing the cytotoxic molecules granulysin (GNLY) and/or GZMB (Fig 6B). Of the NKG7^+^ clusters, two CD4^+^ T cell clusters and four CD8^+^ T cell clusters were identified (Fig 6B and C). The two NKG7^+^ CD4^+^ T cell clusters (C05 and C20) appeared to be effector memory cell clusters as both had minimal CD27 and CD45RA expression (Fig 6B), and cluster 20 having high CD57 expression (Fig 6C). Three of the NKG7^+^ CD8^+^ T cell clusters were grouped together in the UMAP (C11, C17 and C18), with cluster 11 and cluster 17 overlapping, suggesting phenotypic and possible functional similarities (Fig 6A). Cluster 13 was clearly separated from the other three NKG7^+^ CD8^+^ T cells clusters in the UMAP (Fig 6A) and had high CD161 expression (Fig 6B and C), overlapping several NKG7^+^ CD8^+^ T cells clusters observed in CHMI samples (Fig 5).

By using the same analysis methods employed for CHMI samples, the two NKG7^+^ CD4^+^ T cell clusters (C05 and C20) were found to be the only clusters with significant differences in abundance when comparing the acute infections of the two different *Plasmodium* species, with both clusters increased during acute *Pf* infection relative to *Pv* (S4 Fig). When acute *Pf* infection was compared to convalescence, none of the four significantly different clusters included CD4^+^ or CD8^+^ T cells (S5 Fig), although two NKG7-expressing NK cell clusters were increased (clusters 7 and 8; S6 Fig). However, one of the four clusters identified as being significantly increased in frequency in acute *Pv* infection, compared to convalescence, was cluster 11, a NKG7^+^ CD8^+^ T cell cluster, consistent with a role for cytotoxic CD8^+^ T cells in immune responses to *Pv* malaria [47] (S6 Fig).

We also compared differences within the NKG7^+^ CD4^+^ and CD8^+^ T cells clusters between patient groups. In both NKG7^+^ CD4^+^ T cell clusters, there was significantly higher expression of NKG7 expression in acute *Pv* infection, when compared to acute *Pf* infection (*P* < 0.05; Fig 6D). The only other difference between the NKG7^+^ CD4^+^ T cell clusters was CD57 expression, which was much higher overall in cluster 20, although no difference was observed between patient groups. This cluster also had a small increase in the overall expression of cytotoxic molecules, particularly GZMB and GNLY, relative to the other CD4^+^ T cell clusters, cluster 5 (Fig 6B-C). The four NKG7^+^ CD8^+^ T cell clusters also had increased NKG7 expression during acute *Pv* infection when compared to acute *Pf* infection but was only significantly increased in cluster 18 (*P* < 0.01; Fig 6D). Cluster 18 also had significantly higher expression of HLA-DR during *Pv* infection when compared to *Pf* infection (*P* < 0.05; Fig 6E) and overall had higher CD57 expression in both groups when compared to the other clusters. Cluster 13 was a non-cytotoxic cluster and had high levels of CD161 expression, which was significantly increased in acute *Pf* infection, when compared to acute *Pv* infection (*P* < 0.05; Fig 6E). KLRG1 expression in cluster 13 followed the same pattern when the two acute infections were compared (*P* < 0.05; Fig 6E). Overall, this data confirms that the NKG7 expression patterns found in CD4^+^ and CD8^+^ T cells in CHMI samples were similar to those observed in natural infection, with some notable differences between patients infected with different *Plasmodium* species.

## Discussion

CD4^+^ and CD8^+^ T cells play pivotal roles in host immune responses during malaria. They are essential for the killing and clearing of *Plasmodium* parasites and for the development of immunity [3,85]. Recent research has shed light on the involvement of NKG7 in parasitic-causing diseases, including both leishmaniasis and malaria [53]. The study presented here was designed to elucidate the role of NKG7 in CD4^+^ and CD8^+^ T cells during malaria. Further to its known role in facilitating CD8^+^ T cell exocytosis and enhancing the cytotoxic function of CD8^+^ T cells during *Pb*A infection, we also found NKG7 was critical for development of IL-10-producing Tr1 cells and cytotoxic CD4^+^ T cells in this model. Additionally, NKG7 was shown to be associated with protection during non-lethal *Py*17XNL infection, and to contribute to the development of CD4^+^ T helper subsets, including Th1, Tr1, Tfh and cytotoxic CD4^+^ T cells. In human PBMCs obtained from individuals infected with either *P. falciparum* or *P. vivax*, NKG7 was found within cytotoxic cell subsets, and notably, within some distinctive, non-cytotoxic cell populations. Furthermore, we discovered NKG7 species-specific differences in samples from patients with *P. falciparum* and *P. vivax* malaria from Sabah, Malaysia. These findings contribute to our understanding about how the host’s immune system responds to malaria, and highlight the dual potential of targeting NKG7 activity for both stimulating immune responses against intracellular pathogens and applications for dampening inflammation.

The functions of CD8^+^ T cells have been extensively documented in *Plasmodium* infection, particularly regarding their role in mediating protection at the liver stage of disease [85,86]. In agreement with numerous transcriptional studies implicating NKG7 in the function of cytotoxic effector CD8^+^ T cells [63,64,87–90], we have reported a key role for NKG7 in CD8^+^ T cell exocytosis and cytotoxicity. However, this was associated with NKG7-exacerbated parasite accumulation in tissues and the onset of ECM following *Pb*A infection [53]. Thus, *Nkg7*-deficient CD8^+^ T cells exhibit reduced expression of key cytotoxic molecules, such as granzyme B, perforin and CD107a, and a diminished capacity to eliminate target cells. Recent studies in other disease models have confirmed these findings for NKG7, emphasising a decreased killing ability and impaired degranulation in the absence of NKG7 [63,77,91–94]. Of particular interest, in one of these studies [91], structural similarities were observed between the four membrane-spanning domains of NKG7 and the γ subunit of an L-type voltage-gated calcium channel. In this context, the authors were able to demonstrate that reduced NKG7 expression compromised calcium flux in CD8^+^ T cells [91]. This aligns with the hypothesis that NKG7 is involved in regulating important intracellular processes, such as intracellular calcium flux, which are crucial for numerous T cell functions. Together, these findings highlight the potential benefits of increasing NKG7 expression for enhancing cytotoxicity anti-parasitic responses. If NKG7 indeed improves the ability of CD8^+^ T cells to eliminate target cells, then the overexpression of this protein could represent a novel therapeutic strategy with broad applicability in various clinical contexts, including infectious diseases caused by intracellular pathogens, as well as cancer. In fact, a recent study demonstrated gain-of-function (tumour killing) in CAR T cells ectopically over-expressing NKG7 in pre-clinical cancer models [95]. However, the consequences of this on the outcome of immunotherapy, and other approaches to modulate NKG7 would have to be carefully monitored.

Th cell subsets play pivotal roles in the immune response to malaria. Th1 cells are essential for cell-mediated, anti-parasitic responses, while Tfh cells are critical for the development of antigen-specific B cells which produce antibodies required for immunity to malaria [3,12,85,96]. The transcription factor T-BET, a master transcription factor for Th1 cell development, binds to the *NKG7* promoter, increasing the expression of *NKG7*, *PRF1* and *IFNG* [65]. NKG7 has also been implicated in other Th cell subsets, including recent reports of expression by cytotoxic CD4^+^ T cells [60–62,97]. In experimental visceral leishmaniasis, NKG7 was found to be crucial for the activation of STAT4 and Th1 cell development [53]. Here, a protective role for NKG7 during *Py*17XNL infection was shown, as well as its involvement in the development and functions of CD4^+^ T cell subsets, including Th1, Tr1 and Tfh cells. NKG7 expression within CD4^+^ T cell subsets was most prominent in Tr1 cells, followed by Th1 cells, with minimal numbers of Tfh cells showing expression. The expression of NKG7 by Th1 and Tr1 cells is supported by previous studies [65–67,98–100]. Additional experiments to address why NKG7 expression is higher in Tr1 cells than in Th1 cells are warranted. A potential explanation is that Tr1 cells represent more terminally differentiated Th1 cells [85], and this may result in the accumulation of NKG7 in this more differentiated cell subset. Alternatively, there may be specific signalling pathways involved in Tr1 cell differentiation that require NKG7. Hence, NKG7 may play an important role in balancing the anti-parasitic functions of Th1 cells with the tissue-protective roles of Tr1 cells, both of which are crucial for mounting an effective immune response during the blood stage malaria. The findings reported here suggest a dual role for NKG7 in both promoting anti-parasitic responses and dampening inflammation in order to protect tissue.

NKG7 has been recognised as a biomarker in various human diseases [74–76,101–105], including different types of cancer [69–73,106,107]. Several human transcriptional studies have associated NKG7 with cytotoxic functions in CD4^+^ [60–62,97] and CD8^+^ T cells [63,64,87–90]. Nevertheless, there has been a lack of comprehensive characterisation of NKG7 at the protein level in human diseases. Here, consistent with previous data, NKG7 was shown to be highly expressed by human cytotoxic and non-cytotoxic PBMCs from participants in CHMI caused by *P. falciparum* infection and uncomplicated malaria patients from disease endemic areas. NKG7 was expressed by populations of CD4^+^ and CD8^+^ cytotoxic T cells, as well as non-cytotoxic T cells. Notably, CD161 was associated with non-cytotoxic CD4^+^ and CD8^+^ T cells that also expressed high levels of NKG7, warranting further investigation. Additionally, *Plasmodium* species-specific differences in NKG7 expression were identified in patients with malaria. Further investigation is required to determine whether these NKG7-specific expression patterns are linked to specific outcomes, such as altered inflammation or immunity. Overall, these findings confirm that NKG7 is expressed by a range of CD4^+^ and CD8^+^ T cell populations in malaria, suggesting a variety of immunological roles following infection that are disease- and context-dependent.

There are several limitations with this study. Although experimental mouse models provide valuable insights, conclusions drawn from them cannot be directly extrapolated to the outcomes of malaria in humans. While certain similarities exist between rodent models or experimental human infection, and clinical infection, exact comparisons are not feasible, so care must be taken when making inferences. Furthermore, we have not directly demonstrated a role for CD4^+^ T cell NKG7 for host protective immunity. Although, we recently generated *Nkg7*-floxed mice that could be used for these studies and made them publically available (C57BL/6J-*Nkg7*^em2Wehi/^J; JAX strain # 039943). Another limitation with the human samples examined was that they were restricted to cases of pre-symptomatic or uncomplicated malaria. It is also important to note that the CHMI datasets involved malaria-naïve volunteers, and outcomes in patients with malaria in disease endemic areas likely differ. Finally, all human datasets were constrained by samples size, limiting statistical power. Thus, careful consideration is required when drawing conclusions, and further investigation with larger samples sizes is warranted.

Overall, the findings in this study highlight the importance of NKG7 expressed by CD4^+^ and CD8^+^ T cells in both malaria disease and immunity, with the outcome influenced by a range of factors including the specific *Plasmodium* species responsible for the infection, the disease context and the organs involved. These data advance our understanding of host immune responses to malaria and suggest that NKG7 may hold promise as a potential therapeutic target to improve immune responses during malaria, as well as other parasitic diseases and inflammatory conditions.

## Materials and methods

### Mice and human ethics

***Mice.*** Experimental mice use was in accordance with the “Australian Code of Practice for the Care and Use of Animals for Scientific Purposes” (Australian National Health and Medical Research Council) and approved by the Queensland Institute of Medical Research (QIMR) Berghofer Medical Research Institute Animal Ethics Committee (AEC; AEC reference number P2304; approval number: A1707-615M).

***CHMI subjects.*** Human ethics approval was provided by the QIMR Berghofer Medical Research Institute Human Research Ethics Committee (HREC; HREC reference number P1479). Written informed consent was received from all participants [82].

***Endemic country malaria patients.*** Ethics approval for the use of human samples was obtained from the QIMR Berghofer Human Research Ethics Committee (HREC reference number P3444), the Northern Territory Department of Health and Menzies School of Health Research ethics committee (HREC 2010–1431), and Medical Research and Ethics Committee, Ministry of Health, Malaysia (NMRR-10-754-6684 and NMRR-12-499-1203). Written informed consent was obtained from all adult study participants or, in the case of children, parents or legal guardians. The inclusion criteria included being 12 years of age or older.

### Experimental mice and human participant details

***Mice.*** The experiments involved the use of female mice aged six week or older, unless otherwise specified. Mice were kept in group housing, with a maximum of six mice per cage. They were maintained in a pathogen-free environment at the QIMR Berghofer Medical Research Institute Animal Facility (Brisbane, Queensland, Australia). B6.*Cd45.1* (Ptprca) mice were obtained from the Animal Resources Centre (Perth, Western Australia, Australia). *C57BL/6J* (WT) mice were obtained from the Walter and Eliza Hall Institute (Melbourne, Victoria, Australia). All other mice were bred at QIMR, including B6J.*Nkg7*^–/–^, PbT-II, PbT-I and *Nkg7*-reporter (*Nkg7*-cre (C57BL/6J-*Nkg7*^em1(cre)Wehi/^J; JAX stock #040147) × mT/mG) lines. Transgenic PbT-II mice [78] were bred with B6J.*Nkg7*^–/–^ mice to produce *Nkg7*-deficient PbT-II mice (PbT-II^Δ*Nkg7*^; CD45.2^+^ CD45.1^–^) mice, and bred with B6.*Cd45.1* mice to produce PbT-II × B6.*Cd45.1* (PbT-II^WT^; CD45.1^+^ CD45.2^+^). Transgenic PbT-I mice [108] were bred with B6J.*Nkg7*^–/–^ mice to produce *Nkg7*-deficient PbT-I mice (PbT-I^Δ*Nkg7*^; CD45.1^–^ CD45.2^+^) mice, and bred with B6.*Cd45.1* mice to produce PbT-I × B6.*Cd45.1* (PbT-I^WT^; CD45.2^+^ CD45.1^+^).

***CHMI subjects.*** PBMCs were obtained from volunteers who participated in a CHMI study involving *P. falciparum*. The primary investigation was a Phase 1b trial conducted at a single centre [82]. The trial aimed to evaluate the safety, tolerability, pharmacokinetic profile, and antimalarial activity of single doses of co-administered artefenomel and piperaquine phosphate against early *P. falciparum* blood-stage infection in healthy adult volunteers. Here, PBMCs from participants who consented for involvement in a study to analyse immunological responses were used. This included 8 individuals, 5/8 male, aged median 22, range 18–29.

***Endemic country malaria patients.*** Blood was obtained from patients enrolled in a comparative clinical-pathophysiological study conducted at Queen Elizabeth Hospital (QEH) in Kota Kinabula, Sabah, Malaysia, spanning from September 2010 to October 2011 [109,110]. This study incorporates analysis of PBMCs obtained from patients recruited in the primary study, including 5 individuals diagnosed with uncomplicated *P. falciparum* (2/5 male, median age 39, range 36–54) and 6 with uncomplicated *P. vivax* malaria (4/6 male, median age 42, range 16–54).

***Plasmodium infection in mice.*** *Plasmodium* infections in mice were established using parasites that had undergone a single passage in C57BL/6J mice. 200 μl of transgenic *Pb*A (231c11) parasites, which were an in-house laboratory stock stored at −80 °C and expressing luciferase and GFP, were thawed at room temperature (RT). These parasites were then injected into a passage mouse via an intraperitoneal (i.p.) route. *Py*17XNL stabilates (in-house laboratory stock, frozen at −80 °C) were washed in NaCl and resuspended in 1 ml sterile PBS (1x) and 200 µl was injected via intravenous (i.v.) route into a passage mouse. Both the *Pb*A and *Py*17XNL passage mice were monitored with parasitemia checks from day 3 post infection (p.i.) and sacrificed when >1% pRBCs. The passage mouse was euthanised and blood was obtained through cardiac puncture and diluted into RPMI/PS (containing 1 IU/ml heparin). The collected blood was then spun at 290 *g* for 7 min at RT. A hemocytometer (Pacific Laboratory Products) was used to enumerate RBCs. Parasite preparations were made, each containin*g* 5 × 10^5^ pRBCs/ml. Subsequently, experimental mice were injected via i.v. route with 200 μl of this preparation, providing approximately 1 × 10^5^ pRBCs per mouse.

***Quantification of Plasmodium parasitemia in mice.*** Passage and experimental mice were tail bled to collect two drops of blood into 250 μl RPMI/PS containing 1 IU/ml heparin. Next, 25 µl of this solution was incubated with SYTO 84 (Invitrogen; Life Technologies) and Hoechst 33342 (Sigma–Aldrich) in RPMI/PS for 22 min at RT. Then, each sample was quenched with 300 µl of RPMI/PS and acquired on a BD LSRFortessa through BD FlowJo version 10.0 (BD Biosciences).

***Preparation of spleen single-cell suspensions.*** Mice were euthanized and a mid-sagittal incision was performed on the abdominal cavity and the spleen was carefully removed. The spleen’s weight was recorded, and it was then placed into 1% fetal bovine serum (FBS) in PBS (1% FBS/PBS). To isolate the cells, the spleens were mechanically mashed using the rear end of a 5 ml syringe plunger (Terumo Medical) through a 100 μm cell strainer (EASYstrainer; Greiner Bio-One). The resulting cell suspension was resuspended in 1% FBS/PBS. Using an Eppendorf Centrifuge 5810R (Thermo Fisher Scientific), the cell suspension was then subsequently centrifuged at 350 *g*. To remove RBCs, the cell pellet was lysed by treating and agitating for 7 min at RT in Red Blood Cell Lysing Buffer Hybri-Max (Sigma–Aldrich). For enumeration, the cells were diluted in Trypan Blue Stain (Invitrogen) and 1x DPBS (Gibco). Further method details included in [53].

***Conventional and spectral flow cytometry.*** Flow cytometry staining protocols were carried out using Falcon 96-Well Clear Round Bottom Tissue Culture-Treated Cell Culture Microplates (Corning). For murine pre-stains, samples were incubated in 30 µl of complete media containing fluorescence-conjugated antibodies reactive against surface proteins for 30 min at 37 °C. 30 µl Zombie Aqua Fixable Viability Dye cocktail (BioLegend) or LIVE/DEAD Fixable Blue Dead Cell Stain Kit (Thermo Fisher) and TruStain FcX (BioLegend; anti-mouse CD16/32) were used for 15 min at RT to treat single-cell suspensions. During a 2 h stimulation at 37 °C, staining for CD107a (LAMP-1) was carried out with PMA/ionomycin or treatment with monensin (described later) by including 1 µl anti-Mouse CD107a (clone 1D4B from BioLegend) to 100 µl of the stimulation cocktail. Other PMA stimulations were carried out for a duration of 2.5 to 3 h. After treatment, cells were washed once with 1% FBS/PBS and spun at 575 *g* for 1 min at 4 °C in an Eppendorf Centrifuge 5810R (Thermo Fisher Scientific). Subsequently, 30 µl of a master mix containing antibodies, conjugated with various fluorophores, reactive against surface proteins were used to stain samples for 30 min at 37 °C. After two washes with 1% FBS/PBS staining buffer, samples were treated with 50 µl of fixation buffer for 20 min at RT. Fixation buffers used were from either the BD Cytofix Fixation Buffer Set (BD Biosciences) or the eBioscience Foxp3/Transcription Factor Staining Buffer Set (Thermo Fisher Scientific). Cells were then rinsed two times with wash buffers from the corresponding kits by spinning for 1 min at 953 *g* at RT. After this, cells were treated with 30 µl of a cocktail of antibodies, conjugated with various fluorophores, against intracellular proteins for 45 min at 37 °C. For human staining, 10^6^ PBMCs per participant, per time point, were aliquoted into plates. Samples were cultured with 50 µl of LIVE/DEAD Fixable Blue Dead Cell Stain Kit (diluted in 1x PBS; Thermo Fisher) at RT for 15 min. After this, cells were rinsed with 2% FBS/PBS and spun for 5 min in an Eppendorf Centrifuge 5810R (Thermo Fisher Scientific). Subsequently, 25 µl of a first master mix of antibodies, conjugated with various fluorophores, reactive against surface proteins samples were used to stain samples for 15 min at RT. After this, 50 µl of a second master mix of antibodies, conjugated with various fluorophores, reactive against surface proteins was added to PBMCs for a further 30 min at RT. After two washes with 2% FBS/PBS staining buffer, samples were treated with 100 µl of fixation buffer from the eBioscience Foxp3/Transcription Factor Staining Buffer Set (Thermo Fisher Scientific) for 20 min on ice. Cells were then washed twice with permeabilisation wash buffer. Following this, 50 µl of a master mix of antibodies, conjugated with various fluorophores, reactive against intracellular proteins cells were used to stain samples for 30 min on ice. All staining procedures were conducted in the dark. Prior to acquisition, samples were stored at 4 °C. Samples were acquired on a BD LSRFortessa (BD Biosciences) using BD FACSDiva version 8.0 or Cytek Aurora 5 laser using the SpectroFlo software package version 2.2 (Cytek Biosciences), then analysed on FlowJo version 10 OSX (FlowJo). GraphPad Prism 9 (version 9.4.0; GraphPad Software) was used for graphing and statistical analyses were conducted with statistical significance defined as P ≤ 0.05. Further method details included in [53].

For assessment of apoptosis and cell viability, cells were washed twice with PBS following surface staining, then diluted in 1x Annexing V binding buffer and 5ul of Annexing V-PE added together with 5ul of 7-AAD reagent were added. Cells were incubated for 15 minutes at room temperature, followed by washing once with 1x Annexing V binding buffer and analyzed by flow cytometry within 1 hour of staining.

Mouse flow cytometry markers used to define cell subsets can be found in Tables 1–5.

**Table 1:** Phenotypic flow cytometry markers for mouse datasets (S1 Fig).

Marker	Phenotype	Antibody source	Catalogue number	Marker	Phenotype	Antibody source	Catalogue number
TCRβ	T cell	BD Biosciences	612821	TIGIT	Activation	BioLegend	142106
NK1.1	NK cell	BioLegend	156510	CCR5	Activation	BioLegend	107016
CD4	T cell	BD Biosciences	563790	CD107a	CTL	BioLegend	121610
CD8	T cell	BioLegend	100730	GZMB	CTL	BioLegend	515408
CD45.1	Congenic marker	BioLegend	110706	LAG-3	Activation	BioLegend	125219
CD45.2	Congenic marker	BD Biosciences	564616	PD1	Activation	BioLegend	135240
IFNγ	Cytokine	BD Biosciences	563854	Perforin	CTL	BioLegend	154406
IL-10	Cytokine	BioLegend	505034				

**Table 2:** Phenotypic flow cytometry markers for mouse datasets (Fig 2).

Marker	Phenotype	Antibody source	Catalogue number	Marker	Phenotype	Antibody source	Catalogue number
TCRβ	T cell	BD Biosciences	612821	PD1	Activation	BioLegend	135218
CD8	T cell	BioLegend	100730	TBET	Th1 cell	BioLegend	644824
CXCR5	Tfh cell	BioLegend	145506	IFNγ	Cytokine	BD Biosciences	563854
BCL6	Tfh cell	BD Biosciences	563581	IL-10	Cytokine	BioLegend	505034
				NK1.1	NK cell	BioLegend	156510

**Table 3:** Phenotypic flow cytometry markers for mouse datasets (Fig 3).

Marker	Phenotype	Antibody source	Catalogue number	Marker	Phenotype	Antibody source	Catalogue number
TCRβ	T cell	BD Biosciences	612821	PD1	Activation	BioLegend	135218
NK1.1	NK cell	BioLegend	156510	eGFP	*Nkg7* expression		
CD4	T cell	BioLegend	100548	Td-Tomato	No *Nkg7* expression		
CD8	T cell	BioLegend	100730	IL-10	Cytokine	BioLegend	505034
FOXP3	Treg cell	BioLegend	126408	CXCR5	Tfh cell	BioLegend	145506
CD45	Lymphocytes	BD Biosciences	564279	CXCR6	Th1	BioLegend	151119
TBET	Th1 cell	BioLegend	644824	PD-1	Activation	BioLegend	135218
IFNγ	Cytokine	BD Biosciences	563854				

**Table 4:** Phenotypic flow cytometry markers for mouse datasets (Fig 4).

Marker	Phenotype	Antibody source	Catalogue number	Marker	Phenotype	Antibody source	Catalogue number
TCRβ	T cell	BD Biosciences	612821	PD1	Activation	BioLegend	135218
NK1.1	NK cell	BioLegend	156510	IFNγ	Cytokine	BD Biosciences	563854
CD4	T cell	BioLegend	100548	IL-10	Cytokine	BioLegend	505034
CD8	T cell	BioLegend	100730	CXCR5	Tfh cell	BioLegend	145506
FOXP3	Treg	BioLegend	126408	CXCR6	Th1 cell	BioLegend	151104
CD45.1	Congenic marker	BioLegend	110706	TBET	Th1 cell	BioLegend	644824
CD45.2	Congenic marker	BD Biosciences	564616				

**Table 5:** Phenotypic flow cytometry markers for apoptosis, activation and proliferation markers (S2 Fig).

Marker	Phenotype	Antibody source	Catalogue number	Marker	Phenotype	Antibody source	Catalogue number
TCRβ	T cell	BD Biosciences	612821	CD11a	Activation	BioLegend	101106
NK1.1	NK cell	BioLegend	156510	CD25	Activation	BioLegend	102008
CD4	T cell	BD Biosciences	563790	CD49d	Activation	BioLegend	103618
CD8	T cell	BioLegend	100730	CD69	Activation	BioLegend	104530
CD45.1	Congenic marker	BioLegend	110706	CellTrace Violet Cell Proliferation Kit	Proliferation	Thermo Fisher	C334557
CD45.2	Congenic marker	BD Biosciences	564616				
Annexin V	Apoptosis, survival	BD Biosciences	559763				
7-AAD	Apoptosis, survival	BioLegend	420404				

Human flow cytometry markers used to define cell subsets can be found in Tables 6 and 7.

**Table 6:** Phenotypic flow cytometry markers for human datasets (Figs 5 and 6).

Marker	Phenotype	Antibody source	Catalogue number	Marker	Phenotype	Antibody source	Catalogue number
CD16	Monocyte	BD Biosciences	612944	CXCR3	Th1 cell	BD Biosciences	565223
CD56	NK cell	BioLegend	318348	CD8	T cell	BioLegend	344734
GZMB	CTL	BioLegend	515408	HLA-DR	Activation	BD Biosciences	335796
CD107a	CTL	BD Biosciences	562622	CD14	Monocyte	BD Biosciences	612902
KLRG1	CTL	Thermo Fisher	46-9488-42	CD49b	Tr1 cell	Thermo Fisher	11-0498-42
CD4	T cell	BD Biosciences	612913	CD38	Activation	BD Biosciences	563964
CD25	Treg cell	BioLegend	356146	CD45RA	TEMRA, Naive	BioLegend	304134
CCR4	Th2 cell	BioLegend	359416	CCR7	TCM, Naive	BD Biosciences	561144
PD1	Activation	BD Biosciences	561272	CCR6	Th17 cell	BD Biosciences	563922
CD3	T cell	Thermo Fisher	58-0032-82	CXCR5	Tfh cell	BioLegend	356942
CD127	Treg cell	BD Biosciences	565185	CD19	B cell	BioLegend	302274
NKG7	NKG7	Beckman-Coulter	IM3293				

**Table 7:** Cytotoxic flow cytometry markers for endemic dataset (Fig 6 and S4 Fig).

Marker	Phenotype	Antibody source	Catalogue number	Marker	Phenotype	Antibody source	Catalogue number
CD3	T cell	BD Biosciences	612893	CD4	T cell	BD Biosciences	612912
GNLY	CTL	BD Biosciences	558254	CD127	MPEC	BioLegend	351308
GZMB	CTL	BioLegend	372204	HLA-DR	Activation	BioLegend	307672
NKG7	NKG7	Beckman-Coulter	IM3293	CD16	NK cell	BD Biosciences	563785
CD45RA	TEMRA, Naive	BioLegend	304138	TCRγδ	T cell	BD Biosciences	564157
CD27	TCM, Naive	BioLegend	356418	PRF1	CTL	BD Biosciences	563763
CD8	T cell	BioLegend	344740	TCRVδ2	T cell	BioLegend	331420
CD56	NK cell	BD Biosciences	612766	KLRG1	SLEC	Thermo Fisher	25-9488-42
CD25	Activation	BD Biosciences	565106	CD161	Activation	BD Biosciences	563864
CD57	Differentiation	BioLegend	359608				

***PMA/ionomycin restimulation.*** Cells were cultured in complete media supplemented with 25 ng/ml PMA (Sigma–Aldrich) and 1 μg/ml (1.33 nM) ionomycin calcium salt (Sigma–Aldrich), with the addition of 2x monensin solution (BioLegend). The PMA/ionomycin restimulation process was carried out over a 2–3 h duration at 37 °C in the presence of 5% CO_2_. Further method details included in [53].

***Isolation of PbT-II and PbT-I cells by FACS.*** PbT-II cells were enriched prior to FACS by using either the CD4 + T cell Isolation Kit, mouse (Miltenyi Biotec) or EasySep Mouse CD4 + T Cell Isolation Kit (STEMCELL) following the manufacturer’s guidelines. PbT-I cells were enriched prior to FACS by using either the CD8 + T cell Isolation Kit, mouse (Miltenyi Biotec) or EasySep Mouse CD8 + T Cell Isolation Kit (STEMCELL) following the manufacturer’s guidelines. PbT-II and PbT-I cells were stained for surface proteins prior to isolation by cell sorting for RNA-sequencing and real-time quantitative PCR (RT-qPCR). The surface staining included use of anti-mouse CD90.2 (BV605; 53-2.1), anti-mouse CD45.2 (PE; 104), anti-mouse CD45.1 (FITC; A20) and either anti-mouse CD4 (APC; GK1.5) for PbT-II cells or anti-mouse CD8 (APC; 53-6.7) for PbT-I cells (all from BioLegend). Dead cells were omitted during flow cytometry with a positive stain for SYTOX Blue Dead Cell Stain. Cells were sorted on a BD FACSAria II or BD FACSAria III (Becton Dickinson), treated in buffer RLT (Qiagen) and stored at −80 °C. Furhter method details included in [53].

***Adoptive cell transfer of PbT-II and PbT-I cells.*** PbT-II cells were isolated from the spleens of PbT-II^WT^ (CD45.1 x PbT-II) and PbT-II^Δ*Nkg7*^ mice using either the CD4^+^ T cell Isolation Kit, mouse (Miltenyi Biotec) or EasySep Mouse CD4 + T Cell Isolation Kit (STEMCELL) following the manufacturer’s guidelines. PbT-I cells were isolated from the spleens of PbT-I^WT^ (CD45.1 x PbT-I) and PbT-I^Δ*Nkg7*^ mice using either the CD8a^+^ T Cell Isolation Kit, mouse (Miltenyi Biotec) or EasySep Mouse CD8^+^ T Cell Isolation Kit (STEMCELL) following the manufacturer’s guidelines. PbT-II^WT^ and PbT-II^Δ*Nkg7*^ cells were enumerated and pooled in equal numbers to a final concentration of 1x10^6^ cells/ml. Then, 1 day before infection with *Pb*A or *Py*17XNL, 200 μl (2x10^5^ cells) was injected via i.v. route into the tail vein of B6.*Cd45.1* recipient mice. Similarly, PbT-I^WT^ and PbT-I^Δ*Nkg7*^ cells were enumerated and pooled in equivalent numbers to a final concentration of 5x10^6^ cells/ml. Then, 200 μl (1x10^6^ cells) were injected via i.v. route into the tail vein of B6.*Cd45.1* recipient mice 1 day before infection with *Pb*A. Additional method details included in [53]. For assessment of PbT-II^WT^ and PbT-II^Δ*Nkg7*^ proliferation, cells were loaded with 5uM CTV (Violet Celltrace) for 15 minutes at room temperature and washed once before cell transfer.

***HEK293T transient transfection.*** HEK293T cells were plated at 0.5 x10^6^ in 60mm plate (Falcon) with 2 ml DMEM (Gibco) supplemented with 10% heat inactivated FCS (Gibco) for 48 hours at 37°C 5% C0_2_ or until 50–60% confluent. NKG7-GFP reporter cells were generated by adding a transfection mix of RD114 envelope plasmid (1.5 μg), PeqPam Gag-pol plasmid (2 μg) and vector plasmid eGFP-T2A-NKG7 (6.25 μg) with 19 μl FuGENE 6 transfection reagent (Promega) in a final volume of 470 μl of DMEM, dropwise to the cells with gentle mixing. After transfection cells were incubated for 72 hours before flow cytometry analysis.

***NK92 Cell culture.*** NK92 human NK cell line (Malignant non-Hodgkin’s lymphoma) was cultured in RPM1640 (Gibco) supplemented with 10% heat inactivated FCS (Gibco), 1x Glutamax (Gibco), 100 units/ml Penicillin-Streptomycin (Gibco) and 1000 units/ml recombinant human IL-2 (Miltenyi). Cells were incubated at 37°C 5% C0_2_ and were passaged every 48 hours in 10 ml volume at a density of 2–5 x10^5^ cells/ml.

***Nucleofection and CRISPR Cas9.*** Gene editing was performed on 5 x10^5^ NK92 cells as previously described (Huang RS). Briefly, for CAS9-RNP preparation 2ul of CAS9- NLS (40 μM) (QB3 Macrolab) were mixed with 2.4ul of human *NKG7* sgRNA sequence: CATGGCCACCACCATGGAGA (100 μM) (Synthego) and incubated for 15 minutes at 37°C. NK92 cells were washed once in dPBS (Gibco) and suspended in 20ul of freshly prepared nucleofection buffer containing 150 mM sodium phosphate buffer (pH 7.2), 5 mM KCl, 15 mM MgCl_2_, 15 mM HEPES and 50 mM mannitol (SIGMA), cells were added to the CAS9-RNP construct and 24.4 μl volume was transferred to a 16 well nucleofection strip (Lonza) for pulsing with program CM-137 on the Amaxa 96 well shuttle (Lonza). Nucleofected cells were transferred to a 24 well plate (Falcon). One ml of warm culture media was added and cells were incubated for 96 hours at 37°C 5% C0_2_, followed by flow cytometry staining.

***RNA extraction.*** NK92 cells were preserved in RLT buffer and subsequently homogenised in QIAshredder columns (both from Qiagen). The RNA extraction process was performed using either the RNeasy Mini Kit or RNeasy Micro Kit (both from Qiagen), following the manufacturer’s guidelines. RNA integrity was assessed using the RNA 6000 Pico Kit (Agilent Technologies) (Performed at QIMR Berghofer sequencing facility). Subsequently, the isolated RNA for RT-qPCR was reverse transcribed into cDNA using the iScript cDNA Synthesis Kit (Bio-Rad) following the manufacturer’s instructions.

***RT-qPCR.*** Taqman Gene Expression Assays (Life Technologies) were utilised in conjunction with GoTaq Probe qPCR Master Mix (Promega Corporation) following the recommended cycling settings suggested by the manufacturer. Reactions were carried out in a volume of 10 μl, including 1 μl of template cDNA. RT-qPCR was conducted in HardShell 384-Well Plates (Bio-Rad). Microseal ‘B’ PCR Plate Sealing Film (Bio-Rad) was used to seal the plates. CFX96 Touch Real-Time PCR Detection System (Bio-Rad) was used to run the TaqMan Gene Expression Assays. To determine relative gene expression levels, the comparative CT method was employed, using mean CT values of *NKG7*, compared to *18S* (for TaqMan Gene Expression Assays).

### Statistical analysis

Statistical analysis was performed using GraphPad Prism 6 (GraphPad Software, Boston, MA) and JMP Pro (v19.1.2, SAS Institute, Cary, NC). Analysis of parasite area under the curve (AUC) in mouse experiments was performed using a Mann-Whitney test, while differences in mouse flow cytometry data was tested using a two-way ANOVA with Sidak’s multiple comparisons test or one-way ANOVA with Tukey’s multiple comparisons test, as appropriate. For analysis of PBT-II co-transfer experiments (S2 Fig), a random effects regression model was used to account for the paired measures for each mouse. The model includes Day (post-infection), Condition (PbT-II genotype), and the Day by condition interaction as factors. If the interaction was significant, we then compared the results of the 2 conditions at each day, while accounting for the paired nature of the data. In other mouse experiments, differences in PbTII cell frequencies were assessed using a Wilcoxon test. Analysis of human cell clusters was performed using Edge R, while differences in immune molecule expression by clusters was assessed by one-way ANOVA with Tukey’s multiple comparisons test.

All additional resources and equipment used in this study are listed in Table 8.

**Table 8:** Resources and equipment [111].

REAGENT or RESOURCE	SOURCE	IDENTIFIER
Human TruStain FcX	BioLegend	Cat#422302
TruStain FcX (α-mouse CD16/32) Antibody (clone: 93)	BioLegend	Cat#101320
TruStain FcX PLUS α-mouse CD16/32 (clone: S17011E)	BioLegend	Cat#156604
**Chemicals**		
2-Mercaptoethanol	Sigma-Aldrich, St. Louis MI, USA	Cat#M6250
BD GolgiStop Protein Transport Inhibitor (Containing Monensin)	BD Biosciences	Cat#554724
Benzonase Nuclease		Cat#9025-65-4
Brilliant Stain Buffer Plus	BD Biosciences	Cat#566385
Dulbecco’s Phosphate Buffered Saline (DPBS)	Gibco, Life Technologies, Carlsbad CA, USA	Cat#14190250
Ficoll-Paque PLUS	GE Healthcare, Little Chalfont, Buckinghamshire, U.K.	Cat#17-1440-03
Hoeschst 33342 (bisbenzimide H 333342 trihydrochloride Cas#23491-52-3)	Sigma-Aldrich	Cat#B2261
Ionomycin calcium salt from *Streptomyces conglobatus*	Sigma-Aldrich	Cat#I0634-1MG
MEM Non-Essential Amino Acids Solution (100X)	Gibco, Life Technologies	Cat#11140050
Penicillin-Streptomycin (10,000 U/ml)	Gibco, Life Technologies	Cat#14140122
Phorbol 12-myristate 13-acetate (PMA)	Sigma-Aldrich	Cat#P8139-1MG
Red Blood Cell Lysing Buffer Hybri-Max	Sigma-Aldrich	Cat#R7757
Syto 84 Orange Fluorescent Nucleic Acid Stain	Invitrogen, Carlsbad CA, USA	Cat#S11365
Trypan Blue Stain (0.4%) for use with Countess Automated Cell Counter	Invitrogen	Cat#T10282
UltraPure DNase/RNase-Free Distilled Water	Invitrogen	Cat#10977015
**Commercial Kits**		
7-AAD Cell Viability		
Annexin V PE Apoptosis Detection Kit I	BD Biosciences	Cat#559763
CD4 ^+^ T Cell Isolation Kit, mouse	Miltenyi Biotec	Cat#130-104-454
CD8 ^+^ T Cell Isolation Kit, mouse	Miltenyi Biotec	Cat#130-104-075
EasySep Mouse CD4 ^+^ Cell Isolation Kit	STEMCELL	Cat#19852
EasySep Mouse CD8 ^+^ Cell Isolation Kit	STEMCELL	Cat#19853
eBioscience Foxp3/Transcription Factor Staining Buffer Set	eBioscience, Thermo Fisher Scientific	Cat#00-5523-00
LIVE/DEAD Fixable Blue Dead Cell Stain Kit	Thermo Fisher	Cat#L34962
NEBNext Single Cell/Low Input RNA Library Prep Kit for Illumina	New England BioLabs	Cat#E6420S
RNase-Free DNase Set	QIAGEN	Cat#79254
RNeasy Micro Kit	QIAGEN	Cat#74004
RNeasy Mini Kit	QIAGEN	Cat#74104
SYTOX Blue Dead Cell Stain	Thermo Fisher	Cat#S34857
Zombie Aqua Fixable Viability Kit	BioLegend	Cat#423102
Zombie NIR Fixable Viability Kit	BioLegend	Cat#423106
ZymoScript RT PreMix	Zyma Research	Cat#R3012
**Disposables**		
10cc/ml Syringe	Terumo Medical	Cat#SS-10S
1cc/ml Tuberculin Syringe	Terumo Medical, Somerset NJ, USA	Cat#SS-01T
26G x ½” Needle	Terumo Medical	Cat#NN-2613R
5cc/ml Syringe	Terumo Medical	Cat#SS-05S
BD Vacutainer Lithium Heparin (LH) 170 I.U. Plus Blood Collection Tubes	BD Biosciences	Cat#367526
Corning cells strainer, size 100um	Sigma-Aldrich	Cat#CLS43175
Countess Cell Counting Chamber Slides	Invitrogen	Cat#C10228
EASYstrainer 100um	Greiner Bio-One, Kremsmunster, Austria	Cat#542000
Falcon 96-well Clear Round Bottom Tissue Culture (TC)-Treated Cell Culture Microplates	Corning Inc., Corning NY, USA	Cat#353077
**Equipment**		
Bio-Rad CFX384 Touch Real-Time PCR Detection System	Bio-Rad	
Countess II FL Automated Cell Counter	Invitrogen	Cat#AMQAP1000
Cytek Aurora	Cytek	Special order research product
Eppendorf Centrifuge	Fisher Scientific Thermo Fisher Scientific	Cat#05–413
FACSARIA II	BD Biosciences	Special order research product
LSRFortessa: 4 laser configuration	BD Biosciences	Special order research product
LSRFortessa: 5 laser configuration	BD Biosciences	Special order research product
Olympus CX31 Biological Microscope	Olympus Life Science, Shinjuku, Tokyo, Japan	Model#CX31
**Experimental Models**		
Mouse: B6.129(Cg)-*Gt(ROSA)26Sor*^*tm4(ACTB-tdTomato,-EGFP)Luo*^*/*J (B6.mTmG)	Liqun Luo, Howard Hughes Medical Institute, Stanford University, Stanford CA, USA	RRID: IMSR_JAX:007676
Mouse: B6.SJL-Ptprc^a^ Pepc^b^/BoyJ (B6.*Cd45.1*)	Animal Resources Centre, Canning Vale, WA, Australia	RRID: IMSR_JAX:002014
Mouse: B6.Tg(*Nkg7*-cre)/J (B6J.*Nkg7*-cre)	Melbourne Advanced Genome Editing Centre (MAGEC), The Walter and Eliza Hall Institute (WEHI), Parkville VIC, Australia	Commissioned
Mouse: B6J.*Nkg7* ko	QIMRB, Brisbane, Australia	RRID: IMSR_KOMP: VG11445-1.1-Vlcg
Mouse: *C57BL.6J*	WEHI, Kew VIC, Australia	RRID: IMSR_JAX:000664
Mouse: Transgenic PbT-I	QIMRB, Brisbane, Australia	Reference [108]
Mouse: Transgenic PbT-II	QIMRB, Brisbane, Australia	Reference [78]
*Plasmodium berghei*: *Pb*A	QIMRB, Brisbane, Australia	Reference [112]
*Plasmodium yoelii*: *Py*17XNL	QIMRB, Brisbane, Australia	
**Media**		
Dulbecco’s Modified Eagle Medium (DMEM), high glucose, pyruvate	Gibco, Life Technologies	Cat#11995073
Fetal Bovine Serum, certified, US origin	Gibco, Life Technologies	Cat#16000044
Roswell Park Memorial Institute Medium (RPMI) 1640 Medium	Gibco, Life Technologies	Cat#11875119
**Oligonucleotides/Recombinant DNA**		
Hs00366585_g1 human NKG7 FAM-MGB TaqMan probe	Thermo Fisher	Cat#4453320
Hs03003631_g1 Human 18S VIC-MGB TaqMan probe	Thermo Fisher	Cat#4453320
**Software and Packages**		
EdgeR	https://bioconductor.org/packages/release/bioc/html/edgeR.html	
FlowJo, v10 OSX	FlowJo, LLC. https://www.flowjo.com	
Ggplot2	https://www.gsea-msigdb.org/gsea/index.jsp	
GraphPad Prism 9, v9.4.0	GraphPad Software https://www.graphpad.com	
Morpheus	https://software.broadinstitute.org/morpheus/	
OMIQ	OMIQ, by Dotmatics https://www.omiq.ai	
RStudio Desktop	https://postit.co	
The R Project for Statistical Computing	https://www.r-project.org	RRID: SCR_001905

## Supporting information

S1 Fig NKG7 has a limited role in the development of FoxP3^+^ CD4^+^ T cells during *Plasmodium yoelii* 17XNL (*Py*17XNL) infection. (A) C57BL/6 and B6.*Nkg7*^-/-^ (B6^∆*Nkg7*^) mice were infected with non-lethal *Py*17XNL. Layout for the experimental timeline. Created in BioRender. Engwerda, C. (2026) https://BioRender.com/wxzptf9. (B) The gating strategy is shown, as well as IFNγ and IL-10 production by FoxP3^+^ and FoxP3^-^ CD4^+^ T cells after stimulation with phorbol 12 myristate 13-acetate and ionomycin. (C) Frequencies and numbers of FoxP3^+^ CD4^+^ T (Treg) cells at days 7 and 14 post-infection, as indicated. (D) Frequencies of IFNγ- and IL-10-positive Treg and FoxP3-negative CD4^+^ T (conventional; cCD4) cells at timepoints assessed, as indicated. Data are from one experiment (n = 6 mice per group, n = 4 naïve controls per group). A Wilcoxon test was used to test for statistical significance between C57BL/6 andB6^∆*Nkg7*^ mice, but no differences were found (P > 0.05).
(TIF)

S2 Fig NKG7 has a cell-intrinsic role in the development of CD4^+^ T cell responses at day five of *Plasmodium berghei* ANKA (*Pb*A) infection. (A) Congenic *Ptprca* (CD45.1) mice co-transferred with PbT-II^WT^ and PbT-II^Δ*Nkg7*^ cells were infected with *Pb*A, causing experimental cerebral malaria (ECM). Schematic showing the experimental timeline. Created in BioRender. Engwerda, C. (2026) https://BioRender.com/wxzptf9. (B) The gating strategy used for the experiment. (C) The four graphs indicate frequencies of IFNγ^+^, Tr1 (IFNγ^+^ IL-10^+^), Granzyme B^+^ and CD107a^+^ PbT-II^WT^ and PbT-II^Δ*Nkg7*^ cells at the onset of ECM (day 5) after stimulation with phorbal 12-myristate 13-acetate (PMA) and ionomycin *ex vivo*, in the presence of monensin. Data is pooled from two independent experiments. A Wilcoxon test was used to determine statistical significance, where * P < 0.05, *** P < 0.001. n = 14 infected mice. (D) The SPICE graphs depict differences in co-expression of cellular markers between co-transferred transgenic PbT-II^WT^ and PbT-II^Δ*Nkg7*^ cells in the spleen of infected mice (at ECM onset (day 5)) after treatment with phorbal 12-myristate 13-acetate (PMA) and ionomycin, in the presence of monensin. Data is from one experiment. n = 6 infected mice. (E) The graph depicts the frequencies of Perforin^+^ PbT-II^WT^ and PbT-II^Δ*Nkg7*^ cells in the spleen of infected mice (at peak ECM being day 5 p.i.) after treatment with phorbal 12-myristate 13-acetate (PMA) and ionomycin, in the presence of monensin. A Wilcoxon test was utilised to calculate statistical significance, where * P < 0.05. Data represents one experiment. n = 6 infected mice.
(TIF)

S3 Fig Cell intrinsic roles for NKG7 in CD4^+^ T cell activation, proliferation and apoptosis during experimental cerebral malaria during *Plasmodium berghei* ANKA (*Pb*A) infection. (A) Congenic *Ptprca* (CD45.1) mice co-transferred with CTV-labelled transgenic PbT-II^WT^ and PbT-II^Δ*Nkg7*^ cells were infected with *P. berghei* ANKA. Schematic showing the experimental outline. Created in BioRender. Engwerda, C. (2026) https://BioRender.com/wxzptf9. (B) The gating strategy for the experiment. (C) The graph shows the frequencies of PbT-II^WT^ and PbT-II*^ΔNkg7^* cells as a percentage of TCRβ^+^ CD4^+^ T cells in the spleen of mice at daily time points. Data represents two independent experiments. n = 3 naïve and infected mice at each time point. (D) The left representative plot depicts gating for six rounds of cell division for PbT-II^WT^ and PbT-II^Δ*Nkg7*^ cells at day 3 post infection (p.i.). The graph to the right enumerates CTV dilution at day 3 p.i. for each round of cell division. Data represents two independent experiments. n = 3 naïve and infected mice. (E) The representative plots to the left show gating for CD11a, CD25, CD69 and CD49d expression. The graphs to the right depict the frequencies of CD11a^+^, CD25^+^, CD69^+^ and CD49d^+^ PbT-II^WT^ and PbT-II^Δ*Nkg7*^ cells at daily time points p.i. Data represents two independent experiments. n = 3 naïve and infected mice at each time point. (F) The representative plots to the left show gating for Annexin V and 7AAD expression. Cells undergoing early apoptosis are PE-Annexin V positive and 7-AAD negative, while cells in late-stage apoptosis or already dead are PE-Annexin V positive and 7-AAD positive. The graphs to the right depict the frequencies of Annexin V^+^ 7-AAD- and Annexin V^+^ 7-AAD + PbT-II^WT^ and PbT-II^Δ*Nkg7*^ cells at daily time points p.i. Data represents two independent experiments. n = 3 naïve and infected mice at each time point. Significance between paired samples at individual time points was assessed using random effects regression incorporating a paired t-test when interaction terms (day p.i. and PbT-II cell genotype) were statistically significant (P < 0.05), where *** P < 0.001 and * P < 0.05.
(TIF)

S4 Fig The 2G9 monoclonal antibody recognises human NKG7. (A) HEK293 cells were transiently transfected with human NKG7 fused to green fluorescent protein. Flow cytometry analysis of cells showing GFP expression and 2G9 binding (lower panel), compared with no antibody stain (upper panel). Percentage of cells are indicated in each quadrant. (B) The workflow for *NKG7* gene editing with single guide (sg)RNA and CRISPR/Cas9 in the human NK92 cell line. Created in BioRender. Engwerda, C. (2026) https://BioRender.com/wxzptf9. (C) 2G9 binding was assessed by flow cytometry in control NK92 cells (left panel) and *NKG7* edited cells (right panel) with percentages of positive and negative cells indicated. (D) *NKG7* gene expression, relative to 18S RNA, was assessed by qPCR in control NK92 cells and *NKG7* edited cells, as indicated. Significance was assessed using an unpaired t-test, where **** P < 0.001.
(TIF)

S5 Fig NKG7-positive CD4^+^ T cells had an activated phenotype at day 15 post *Plasmodium falciparum* infection. (A) Peripheral blood mononuclear cells were acquired by spectral flow cytometry and analysed with dimensionality reduction using unsupervised FLOWSOM clustering in OMIQ software. PBMCs were divided into 30 clusters and clusters that were significantly different in abundance between day 8 and 15, day 8 and end of study (EOS), and day 15 and EOS are indicated with red circles. Day 0 (n = 8), day 8 (n = 8), day 15 (n = 8), EOS (n = 7). Significance assessed using edgeR. (B) The expression of CD38, Killer cell lectin-like receptor G1 (KLRG1), NKG7, programmed cell death protein 1 (PD1), CD107a, HLA-DR and Granzyme B (GZMB) was measured, and the mean fluorescence intensity (MFI) of cellular markers is shown. Day 0 (n = 8), day 8 (n = 8), day 15 (n = 8), end of study (EOS; n = 7). ** P < 0.01, *** P < 0.001, **** P < 0.0001; significance assessed by one-way ANOVA with Tukey’s multiple comparisons test.
(TIF)

S6 Fig NKG7-expresing CD4^+^ T cell clusters were significantly more abundant during acute uncomplicated *Plasmodium vivax* (*Pv*) infection when compared to acute *P. falciparum* (*Pf*) infection. Peripheral blood mononuclear cells were acquired by spectral flow cytometry and analysed with dimensionality reduction using unsupervised FLOWSOM clustering in OMIQ software. PBMCs were divided into 20 clusters and clusters that were significantly different in abundance between Acute *Pf* and Acute *Pv*, Acute *Pf* vs Convalescence and Acute *Pv* vs Convalescence are indicated with red circles. Acute *Pf* (n = 5), acute *Pv* (n = 5), convalescence (n = 8). Significance assessed using edgeR.
(TIF)

S1 Table Source data. (XLSX)
